# The first NINDS/NIBIB consensus meeting to define neuropathological criteria for the diagnosis of chronic traumatic encephalopathy

**DOI:** 10.1007/s00401-015-1515-z

**Published:** 2015-12-14

**Authors:** Ann C. McKee, Nigel J. Cairns, Dennis W. Dickson, Rebecca D. Folkerth, C. Dirk Keene, Irene Litvan, Daniel P. Perl, Thor D. Stein, Jean-Paul Vonsattel, William Stewart, Yorghos Tripodis, John F. Crary, Kevin F. Bieniek, Kristen Dams-O’Connor, Victor E. Alvarez, Wayne A. Gordon, the TBI/CTE group

**Affiliations:** Department of Neurology, Boston University School of Medicine, 72 East Concord Street, Boston, MA 02118 USA; Department of Pathology, Boston University School of Medicine, 72 East Concord Street, Boston, MA 02118 USA; Alzheimer’s Disease Center, CTE Program, Boston University School of Medicine, 72 East Concord Street, Boston, MA 02118 USA; VA Boston Healthcare System, 150 South Huntington Avenue, Boston, 02130 MA USA; Department of Veteran Affairs Medical Center, 200 Springs Road, Bedford, MA 01730 USA; Department of Neurology, Washington University School of Medicine, 660 South Euclid Avenue, Saint Louis, MO 63110 USA; Department of Neuroscience, Mayo Clinic, 4500 San Pablo Road, Jacksonville, FL 32224 USA; Department of Pathology, Brigham and Women’s Hospital, Harvard Medical School, 75 Francis Street, Boston, MA 02115 USA; Department of Pathology, University of Washington School of Medicine, 325 Ninth Avenue, Seattle, WA 98104 USA; Department of Neurosciences, University of California San Diego School of Medicine, 9500 Gilman Drive, La Jolla, CA 92093 USA; Department of Pathology, Center for Neuroscience and Regenerative Medicine, Uniformed Services University of the Health Sciences, 4301 Jones Bridge Road, Bethesda, MD 20814 USA; Taub Institute for Research on Alzheimer’s disease and the Aging Brain, Columbia University Medical Center, 710 West 168th Street, New York, NY 10032 USA; Department of Neuropathology, University of Glasgow Institute of Neuroscience and Psychology and Queen Elizabeth University Hospital, 1345 Govan Road, Glasgow, G51 4TF UK; Department of Biostatistics, Boston University School of Public Health, 801 Massachusetts Avenue, Boston, MA 02118 USA; Department of Pathology, Fishberg Department of Neuroscience, Friedman Brain Institute, Ronald M. Loeb Center for Alzheimer’s Disease, Icahn School of Medicine at Mount Sinai School, One Gustave L. Levy Place, New York, NY 10029 USA; Department of Rehabilitation Medicine, Icahn School of Medicine at Mount Sinai, 3 East 101st Street, New York, NY 10029 USA

**Keywords:** Chronic traumatic encephalopathy, Traumatic brain injury, Tauopathy, Brain trauma, Neurodegenerative disorders

## Abstract

Chronic traumatic encephalopathy (CTE) is a neurodegeneration characterized by the abnormal accumulation of hyperphosphorylated tau protein within the brain. Like many other neurodegenerative conditions, at present, CTE can only be definitively diagnosed by post-mortem examination of brain tissue. As the first part of a series of consensus panels funded by the NINDS/NIBIB to define the neuropathological criteria for CTE, preliminary neuropathological criteria were used by 7 neuropathologists to blindly evaluate 25 cases of various tauopathies, including CTE, Alzheimer’s disease, progressive supranuclear palsy, argyrophilic grain disease, corticobasal degeneration, primary age-related tauopathy, and parkinsonism dementia complex of Guam. The results demonstrated that there was good agreement among the neuropathologists who reviewed the cases (Cohen’s kappa, 0.67) and even better agreement between reviewers and the diagnosis of CTE (Cohen’s kappa, 0.78). Based on these results, the panel defined the pathognomonic lesion of CTE as an accumulation of abnormal hyperphosphorylated tau (p-tau) in neurons and astroglia distributed around small blood vessels at the depths of cortical sulci and in an irregular pattern. The group also defined supportive but non-specific p-tau-immunoreactive features of CTE as: pretangles and NFTs affecting superficial layers (layers II–III) of cerebral cortex; pretangles, NFTs or extracellular tangles in CA2 and pretangles and proximal dendritic swellings in CA4 of the hippocampus; neuronal and astrocytic aggregates in subcortical nuclei; thorn-shaped astrocytes at the glial limitans of the subpial and periventricular regions; and large grain-like and dot-like structures. Supportive non-p-tau pathologies include TDP-43 immunoreactive neuronal cytoplasmic inclusions and dot-like structures in the hippocampus, anteromedial temporal cortex and amygdala. The panel also recommended a minimum blocking and staining scheme for pathological evaluation and made recommendations for future study. This study provides the first step towards the development of validated neuropathological criteria for CTE and will pave the way towards future clinical and mechanistic studies.

**Electronic supplementary material:**

The online version of this article (doi:10.1007/s00401-015-1515-z) contains supplementary material, which is available to authorized users.

## Introduction

In 1928, the pathologist and medical examiner, Harrison Stanford Martland, introduced the term ‘punch-drunk’ to describe the clinical features of a distinct neuropsychiatric syndrome that affected boxers [26]; a condition that later came to be known as ‘dementia pugilistica’ [33]. Case reports and small series describing the neuropathologic features of the condition appeared in the 1950s and 1960s [3, 6, 16, 27, 35, 41]. Although the histological techniques varied, the most common pathological findings were cerebral atrophy, neuronal loss, gliosis and argyrophilic neurofibrillary tangles. In the seminal 1973 monograph on the clinicopathological features of dementia pugilistica in 15 former male boxers, Corsellis, Bruton, and Freeman-Browne described cerebral atrophy, enlargement of the lateral and third ventricles, thinning of the corpus callosum, cavum septum pellucidum with fenestrations, cerebellar scarring, and argyrophilic neurofibrillary degeneration using cresyl violet and Von Braunmühl’s silver stains [5]. Subsequent re-examination of Corsellis’ original series of boxers and additional cases using beta-amyloid (Aβ) immunohistochemistry determined that 95 % of CTE cases showed widespread diffuse Aβ deposits [43, 46].

Over the following decades, it was recognized that the condition affected men and women with a broad range of exposure to brain trauma, including physical abuse [42], head-banging [13, 18], poorly controlled epilepsy, “dwarf-throwing” [48], and rugby^1^ [13]. Eventually, the term “chronic traumatic encephalopathy” or “CTE”, introduced by Critchley in 1949 [8], became the preferred designation for the condition.

Coincident with the use of more refined methodology, the early pathology of CTE was reported in several young subjects [13, 14, 18]. Hof reported a single case of repetitive head-banging in a young autistic patient with numerous perivascular clusters of thioflavin and Gallyas-positive neurofibrillary tangles (NFTs) and neurites at the depths of the cerebral sulci and in the superficial layers of the inferior temporal, entorhinal and perirhinal cortices in the absence of Aβ plaques [18]. Hof and colleagues also quantitatively demonstrated the preferential distribution of the NFTs in superficial layers II and III in CTE, a laminar predilection characteristic of two other environmentally acquired tauopathies, post-encephalitic parkinsonism and Guamanian parkinsonism dementia complex (GPDC), but not found in Alzheimer’s disease (AD) [19]. Geddes and colleagues further described argyrophilic, hyperphosphorylated tau (p-tau) immunopositive neocortical NFTs and neuropil threads strikingly arranged in groups around small cortical blood vessels, in addition to diffuse granular cytoplasmic immunopositivity in some neurons [13]. Geddes also noted that the topography of the p-tau pathology principally involved the depths of sulci and that there was no Aß deposition in the 5 young cases that formed the basis of their manuscript [13].

Omalu and colleagues were the first to report CTE in a professional American football player [38, 39] and a professional wrestler [37]. Recent neuropathological studies have identified CTE in athletes who played soccer, baseball, ice hockey and rugby, as well as in military personnel exposed to explosive blast [15, 29, 31, 32, 36, 45]. P-tau pathology, with some features of CTE, has also been described following exposure to single moderate or severe traumatic brain injury, together with Aβ plaques [20, 44].

In 2013, McKee and colleagues described a spectrum of p-tau pathology in 68 male subjects with a history of exposure to repetitive brain trauma with neuropathological evidence of CTE, ranging in age from 17 to 98 years (mean 59.5 years). In young subjects with the mildest forms of CTE, focal perivascular epicenters of NFTs and astrocytic tangles (ATs) were found clustered at the depths of the cortical sulci; in subjects with severe disease, a profound tauopathy involved widespread brain regions [32]. Other abnormalities encountered in advanced disease included abnormal deposits of phosphorylated TAR DNA-binding protein of 43 kDa (TDP-43) protein that occasionally co-localized with p-tau, varying degrees of Aβ pathology, axonal dystrophy and neuroinflammation [30, 32]. Based on these findings, preliminary criteria for the neuropathological diagnosis of CTE were proposed, as follows:

1. Perivascular foci of p-tau immunoreactive NFTs and ATs in the neocortex
2. Irregular distribution of p-tau immunoreactive NFTs and ATs at the depths of cerebral sulci
3. NFTs in the cerebral cortex located preferentially in the superficial layers (often most pronounced in temporal cortex)
4. Supportive, non-diagnostic features: Clusters of subpial ATs in the cerebral cortex, most pronounced at the sulcal depths.

In March 2013, the National Institutes of Health (NIH), supported by the Foundation for NIH’s Sports Health Research Program with funding from the National Football League (NFL), launched a major effort to define the neuropathological characteristics of CTE. Two projects were initiated on the neuropathology of CTE and the delayed effects of traumatic brain injury. One of the initial objectives was to convene a consensus meeting to define the neuropathological criteria for the diagnosis of CTE. The primary objective for the first meeting was to determine whether CTE was a distinctive tauopathy that could be reliably distinguished from other tauopathies using the preliminary criteria. The study design was modeled after previous successful NIH-sponsored consensus conferences for other tauopathies, specifically progressive supranuclear palsy (PSP) and corticobasal degeneration (CBD) [10, 17, 25].

## Materials and methods

Individuals not directly involved in the pathological evaluation (ACM, VEA, KFB, JFC) selected 25 cases of the various tauopathies. The selected cases were considered to be representative of the disease and of at least moderate disease severity. The cases included 10 recently acquired cases of suspected CTE that were donated as part of the NINDs-funded traumatic brain injury (TBI) brain bank at Boston University School of Medicine (BUSM), including 7 cases with Aβ plaques and 3 cases without Aß plaques. Five cases of AD, Braak stage V-VI, 2 cases of PSP and 2 cases of CBD were selected from the Alzheimer’s Disease Center (ADC) brain bank at BUSM. Two cases of GPDC and 2 cases of argyrophilic grain disease (AGD) were selected from the Alzheimer’s Disease Research Center (ADRC) brain bank at Mayo Clinic-Jacksonville, and 2 cases of primary age-related tauopathy (PART) were selected from the ADRC brain bank at Columbia University. Paraffin-embedded tissue blocks from 12 brain regions from each case were sent to the TBI Brain Bank at BUSM for uniform processing, staining and immunohistochemistry (IHC) (Table 1); 2 of the selected cases were missing the superior temporal block. Sections were stained with Luxol fast blue counterstained with hematoxylin and eosin (LHE) and Bielschowsky silver impregnation; IHC was performed using anti-Aβ42 (Aß 1-42, EMD Millipore, 1:2000, pretreated with 88 % formic acid for 2 min; or Aβ-4G8, Bio Legend, 1:100,000, pretreated with formic acid); anti-p-tau (AT8, Thermo Fisher Scientific/Pierce, 1:2000, pretreated with formic acid) and anti-p-TDP-43 (Anti-TDP-43, phospho, 1:2000, pretreated with formic acid) for a total of 27 slides per case (25 slides in 2 cases). An individual blinded to the origin and identity of the cases (KFB) scanned the 671 glass pathology slides into digital images at the Mayo Clinic Jacksonville using an Aperio scanner (Leica Biosystems, Buffalo Grove, IL). The digitized images were organized into folders labeled with only the case number (#1-25), brain region, stain and IHC and provided to the evaluating neuropathologists on portable hard drives as well as on an online slide-hosting website (Leica Biosystems-Aperio). No clinical or demographic information was provided to the evaluating neuropathologists—including no information regarding the subjects’ age, gender, clinical symptoms, diagnosis or athletic exposure. No information was supplied regarding the gross neuropathological features of the brains. The neuropathologists were given a tauopathy criteria guide that provided the provisional criteria for CTE [32] as well as published criteria for the other tauopathies (See supplementary material for full tauopathy criteria guide) [4, 7, 11, 21, 25, 34, 40, 47]. Although the neuropathologists knew that the selected cases represented presumptive CTE, AD, PSP, CBD, AGD, PART, and GPDC, they did not know how many cases representing each diagnosis were to be evaluated.

**Table 1:** Brain regions evaluated in the case review

Brain region	Stains/IHC				
	LHE	AT8	Aβ42	TDP43	BIEL
Superior frontal (BA 8, 9)	X	X			
Dorsolateral superior frontal (BA 45, 46)	X	X	X		
Caudate nucleus, nucleus accumbens, putamen	X	X			
Temporal pole (BA 38)	X	X			
Superior temporal gyrus (BA 20, 21,22)	X	X		X	
Amygdala, with entorhinal cortex (BA 28)	X	X			
Hippocampus and lateral geniculate nucleus	X	X	X	X	X
Thalamus and mammillary body	X	X			
Cerebellum with dentate nucleus	X	X			

Digitized images of the following microscopic slides were provided to the evaluating neuropathologists on 25 cases of tauopathies including AD, AGD, CBD, CTE, GPDC, PART and PSP. The slides were all uniformly processed by a single laboratory

*Aβ* Beta-amyloid, *AD* Alzheimer’s disease, *AGD* Argyrophilic grain disease, *BA* Brodmann area, *BIEL* Bielschowsky’s silver method, *CTE* Chronic traumatic encephalopathy, *GPDC* Guamanian Parkinson’s dementia complex, *LHE* Luxol fast blue, counterstained with hematoxylin and eosin, *PART* Primary age-related tauopathy, *PSP* Progressive supranuclear palsy

Seven neuropathologists with experience in neurodegenerative diseases, including the tauopathies, participated in the evaluation of the digitized images (NJC, DWD, RDF, CDK, DPP, TDS, JPV). The neuropathologists evaluated the cases independently, at their own pace, and completed an evaluation form that included the pathological diagnosis and a 4-scale level of certainty (1, unsure; 2, possible; 3, probable; 4, definite). After the initial evaluations were sent to BUSM for analysis, the evaluator was provided the gross neuropathological findings and clinical summaries for each case, and asked to reevaluate the diagnosis and provide a second level of conviction. The results of all evaluations were analyzed prior to the face-to-face meeting held on February 25–26, 2015.

### Statistical analysis

To evaluate the agreement among the neuropathologists who reviewed the cases, two sets of Cohen’s kappa statistics were calculated. The first kappa coefficient measured the agreement among the overall neuropathological diagnoses; the second kappa coefficient measured the agreement among neuropathologists regarding the specific diagnosis of CTE. The overall kappa coefficient combines the neuropathologist-level estimates of kappa into an overall estimate of the common agreement. Kappa values of 0.81–1.0 indicate very good agreement, kappa values of 0.61–0.80 show good agreement, while kappa values of 0.41–0.60 indicate moderate agreement [12]. All statistical analyses were done using SAS 9.4 (SAS Institute Inc., Cary, North Carolina, USA) software.

At the face-to-face consensus meeting, a larger panel that included ACM^2^, WS,^2^ IL, WAG and members of the NINDs TBI/CTE group reviewed the results of the neuropathological evaluations, digitized images and glass pathology slides and discussed the cases as a group. Discussions led to refinements in the neuropathological criteria for CTE, as well as “best practice” recommendations for neuropathologists examining brains for evidence of CTE.

## Results

There was good agreement regarding the overall neuropathological diagnosis of all 25 cases (Cohen’s kappa, 0.67), and even better agreement regarding the specific diagnosis of CTE (Cohen’s kappa, 0.78), using the proposed criteria. In evaluating the 10 cases submitted with the presumptive diagnosis of CTE (Supplementary Table 1), 64 of the 70 reviewers responses (91.4 %) indicated CTE as the diagnosis. There was a significant decrease of errors that paralleled the sequence of cases evaluated. The log of the expected errors significantly decreased by 0.43 for each case of CTE reviewed (*p* value = 0.024). There were common additions to the CTE diagnosis, including “Changes of Alzheimer’s disease” (ADC) and AD in the cases with Aß plaques (cases #4–10). Other co-morbid diagnoses included hippocampal sclerosis (HS), AGD and PART. In the 15 other tauopathy cases (cases submitted for review with diagnoses other than CTE) (Supplementary Table 2), the reviewers generally agreed with the submission diagnoses of AD (97.1 % of responses), CBD (92.8 %), and PART (78.5 %); however, there were frequent discrepancies in cases with the presumptive diagnoses of PSP, AGD and GPDC (Supplementary Table 2). The evaluators reported a significantly increased degree of certainty (*t* test = 4.36, *p* value <0.001) in the diagnosis of CTE from an overall mean of 3.1 in a 4-point scale of conviction (1, unsure; 2, possible; 3, probable; 4, definite) to a mean of 3.7 after the gross neuropathological features and clinical features of the cases were provided to the evaluator. Three initial diagnoses of non-CTE were changed to CTE and 9 diagnoses of co-morbid CTE in non-CTE cases were changed to no CTE after revealing the clinical and gross neuropathological features.

### Diagnostic neuropathological features of CTE

**Fig. 1:**
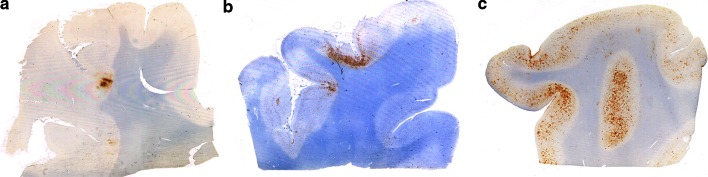
Low magnification inspection of p-tau-stained slides often revealed the irregular spatial pattern of CTE pathology. AT8-stained slides of cerebral cortex in 3 cases of CTE showing irregular patches of p-tau pathology most dense at the depths of the sulci

**Fig. 2:**
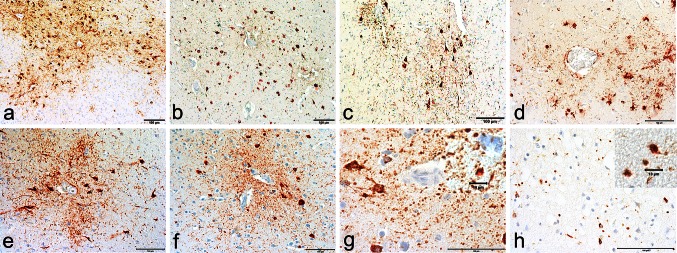
The microscopic features of the pathognomonic lesion of CTE. The pathognomonic feature of CTE is a perivascular accumulation of p-tau aggregates in neurons, astrocytes and cell processes in an irregular spatial pattern in the cerebral cortex and found preferentially at the depths of the sulci. **a** A large perivascular p-tau lesion is found at the sulcal depths in a subject with CTE. **b**–**f** Multiple perivascular foci are often found in the cortex in CTE. **g** The p-tau aggregates in CTE include strikingly rounded structures in the neuropil that often are most dense in the areas surrounding the vessel. **h** The rounded p-tau immunoreactive cell processes are more densely distributed than those found in argyrophilic grain disease. All sections immunostained for AT8, *bars* indicate 100 µm, except in **g** and **h** where the *small bars* indicate 10 µm

The group defined a neuropathological lesion specific to CTE that distinguished the disorder from other tauopathies. The pathognomonic lesion of CTE consists of p-tau aggregates in neurons, astrocytes, and cell processes around small vessels in an irregular pattern at the depths of the cortical sulci (Figs. 1, 2; Table 2). The group also noted that the distinctively irregular spatial pattern of p-tau in CTE was often visible with low-power inspection (Fig. 1). Although other abnormalities in p-tau were also found, especially in the more severely affected brains, the pathognomonic lesion was distinct and not found in the other degenerative tauopathies (Fig. 2). In addition, the group observed frequent evidence of other pathologies in CTE, including TDP-43-immunoreactive neuronal cytoplasmic inclusions, Aβ plaques and amyloid angiopathy, and hippocampal neurofibrillary degeneration, including extracellular tangles best seen with silver stains.

**Fig. 3:**
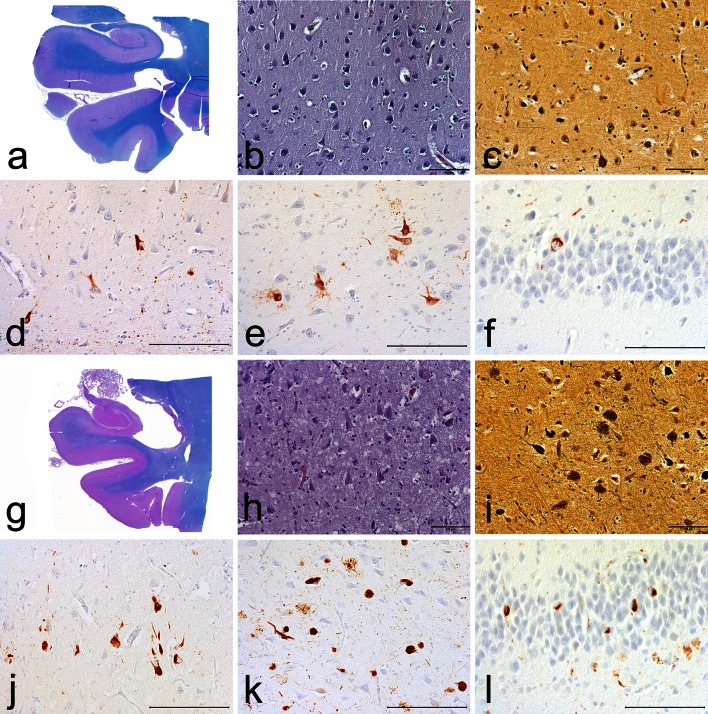
Hippocampal pathology in CTE. Examples of hippocampal pathology in 2 cases of CTE of moderate severity. In example 1 (**a**–**f**), there is **a** mild hippocampal atrophy, **b** mild neuronal loss in CA1, **c** sparse NFTs in CA1, Bielschowsky silver stain, **d** sparse NFTs in CA1, AT8 immunostain, **e** moderate numbers of diffusely immunopositive AT8 stained neurons in CA4, and **f** occasional AT8 immunopositive NFTs in the dentate gyrus. In example 2 (**g**–**l**), there is **g** more severe hippocampal atrophy, **h** clear neuronal loss in CA1, **i** moderate density of NFTs in CA1, Bielschowsky silver stain, **j** moderate density of NFTs in CA1, AT8 immunostain, **k** high numbers of AT8-stained neurons and NFTs in CA4, and **l** moderate numbers of AT8 immunopositive NFTs in the dentate gyrus. *Bars* indicate 100 µm

**Fig. 4:**
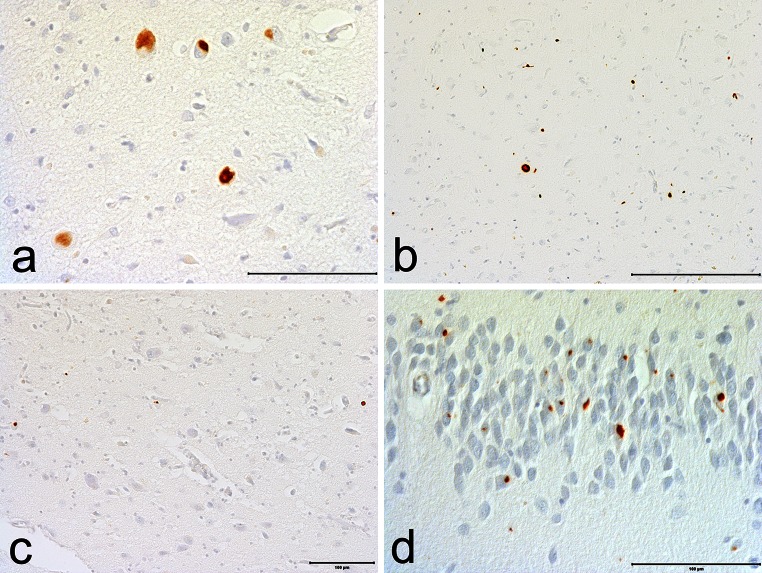
pTDP-43 pathology in CTE. **a** pTDP-43 neuronal inclusions in the amygdala. **b** p-TDP-43 inclusions and dot-like neurites in CA1. **c** p-TDP-43 dot-like neurites in entorhinal cortex. **d** pTDP-43 inclusions and dot-like neurites in the dentate granule cell layer. All sections immunostained for p-TDP-43, bars indicate 100 µm

**Table 2:** Preliminary NINDS criteria for the pathological diagnosis of CTE

Required for diagnosis of CTE	
1.	The pathognomonic lesion consists of p-tau aggregates in neurons, astrocytes, and cell processes around small vessels in an irregular pattern at the depths of the cortical sulci

Supportive neuropathological features of CTE	
p-Tau-related pathologies:	
1.	Abnormal p-tau immunoreactive pretangles and NFTs preferentially affecting superficial layers (layers II–III), in contrast to layers III and V as in AD
2.	In the hippocampus, pretangles, NFTs or extracellular tangles preferentially affecting CA2 and pretangles and prominent proximal dendritic swellings in CA4. These regional p-tau pathologies differ from the preferential involvement of CA1 and subiculum found in AD (Fig. 3)
3.	Abnormal p-tau immunoreactive neuronal and astrocytic aggregates in subcortical nuclei, including the mammillary bodies and other hypothalamic nuclei, amygdala, nucleus accumbens, thalamus, midbrain tegmentum, and isodendritic core (nucleus basalis of Meynert, raphe nuclei, substantia nigra and locus coeruleus)
4.	p-Tau immunoreactive thorny astrocytes at the glial limitans most commonly found in the subpial and periventricular regions
5.	p-Tau immunoreactive large grain-like and dot-like structures (in addition to some threadlike neurites) (Fig. 2h)
Non-p-tau-related pathologies:	
1.	Macroscopic features: disproportionate dilatation of the third ventricle, septal abnormalities, mammillary body atrophy, and contusions or other signs of previous traumatic injury
2.	TDP-43 immunoreactive neuronal cytoplasmic inclusions and dot-like structures in the hippocampus, anteromedial temporal cortex and amygdala (Fig. 4)

Age-related p-tau astrogliopathy that may be present; non-diagnostic and non-supportive [22]	
1.	Patches of thorn-shaped astrocytes in subcortical white matter
2.	Subependymal, periventricular, and perivascular thorn-shaped astrocytes in the mediobasal regions
3.	Thorn-shaped astrocytes in amygdala or hippocampus [22]

### Supportive neuropathological features of CTE

The group defined supportive pathological features for CTE. These features were commonly found in the CTE cases in addition to the required criteria, but were not considered diagnostic in isolation (Table 2).

### Exclusions to the sole diagnosis of CTE

The presence of changes compatible with the diagnosis of another neurodegenerative disease excludes CTE as a single diagnosis, and indicates the presence of co-morbid pathology. These features include CA1-predominant neurofibrillary degeneration in the hippocampus in association with Aβ plaques consistent with AD [34]; prominent cerebellar dentate nucleus cell loss, coiled bodies in oligodendroglia, and tufted astrocytes as seen in PSP [25]; severe involvement of the striatum and pallidum with extensive astrocytic plaques in cortical and subcortical structures as seen in CBD [21] or globular astrocytic inclusions of globular glial tauopathy [1].

## Discussion

The consensus panel of neuropathologists found that the p-tau pathology of CTE is clearly distinct from other tauopathies. The panel concluded that there is a pathognomonic lesion of CTE that consists of an accumulation of abnormal tau in neurons and astroglia distributed around small blood vessels at the depths of sulci in the cortex in an irregular spatial pattern. Other supportive features of CTE include abnormal p-tau immunoreactive pretangles and NFTs preferentially affecting superficial layers (layers II–III), pretangles, NFTs or extracellular tangles primarily in CA2 and CA4 of the hippocampus, NFTs in subcortical nuclei, including the mammillary bodies and other hypothalamic nuclei, amygdala, nucleus accumbens, thalamus, midbrain tegmentum, isodendritic core (nucleus basalis of Meynert, raphe nuclei, substantia nigra and locus coeruleus), p-tau immunoreactive thorned astrocytes at the glial limitans in the subpial and periventricular regions, p-tau immunoreactive large grain-like and dot-like structures, and TDP-43 immunoreactive neuronal cytoplasmic inclusions and dot-like structures in the hippocampus, anteromedial temporal cortex and amygdala. While this was only the first meeting to address the neuropathological diagnosis of CTE, and more research is needed to determine the nature and degree of brain injury necessary to cause this neurodegeneration, the panel members also noted that the pathognomonic lesion of CTE has, thus far, only been found in individuals who were exposed to brain trauma, typically multiple episodes.

**Fig. 5:**
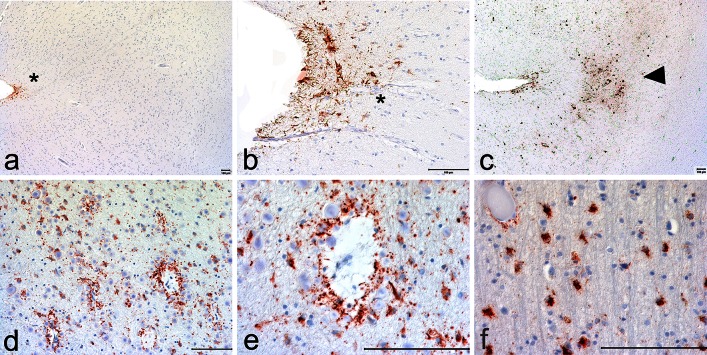
Age-related p-tau astrogliopathy that may be present. **a** and **b**. Subpial p-tau immunopositive astrocytes may be found at the glial limitans in the sulcal depths but are non-specific and non-diagnostic for CTE (*asterisks*). **c** However, p-tau immunopositive subpial astrocytes accompanied by perivascular foci of p-tau positive neurons and astrocytes (*arrowhead*) at the depths of the sulci are diagnostic for CTE. **d** and **e** p-Tau immunopositive astrocytes surrounding small venules in the deep white matter of the temporal lobe are not diagnostic for CTE and are often found in association with aging [22]. **f** p-Tau positive astrocytes may also be found in the crests of the white matter of the frontal and temporal lobes with aging and other conditions that are not diagnostic for CTE [22]. All sections immunostained for AT8, *bars* indicate 100 µm

The panel also determined that the pathognomonic lesion of CTE is distinct from age-related tau astrogliopathy (ARTAG), a morphological spectrum of astroglial pathology detected by p-tau immunohistochemistry that may coexist in the same brain with other disorders and is of unclear etiology (Fig. 5). P-tau-immunoreactive astrocytes in ARTAG include thorn-shaped astrocytes in the subpial, subependymal, and perivascular regions of the white and gray matter (Kovacs, in press). Changes of ARTAG may be present in CTE, but in isolation, are non-specific and non-diagnostic.

Although 9 of the 10 subjects diagnosed with CTE in this study were former American football players and only one was a former professional boxer, previous data has shown that the pathological features of CTE associated with boxing (often referred to as “dementia pugilistica”) are similar to the pathological features of CTE associated with football [28, 32]. Furthermore, the cortical areas most likely to show early focal CTE pathology in boxers are similar to American football players. While initial reports of boxers with CTE described cerebellar scarring, atrophy and loss of Purkinje cells [5], recent studies of pugilists find that cerebellar pathology is rare aside from p-tau NFTs and neurites in the dentate nucleus, Purkinje cells and roof of the 4th ventricle [28, 32].

### Future directions

Using criteria from this consensus meeting, Bieniek and colleagues reviewed the clinical records and brains of 1721 cases donated to the Mayo Clinic Brain Bank over the past 18 years, and found CTE pathology in 32 % of contact sport athletes [2]. No cases of CTE were found in 162 control brains without a history of brain trauma or in 33 cases with a history of a single traumatic brain injury. Of the 21 with CTE pathology, 19 had participated in football or boxing, and many were multiple sport athletes including rugby, wrestling, basketball, and baseball. One athlete played only baseball, and another athlete only played basketball. Similarly, Ling and colleagues screened 268 cases of neurodegenerative disease and controls in the Queen Square Brain Bank for Neurological Disorders using the preliminary McKee criteria [32] and found changes of CTE in 11.9 % of neurodegenerative disorders and 12.8 % of elderly controls. Of the cases with changes of CTE, 93.8 % had a history of TBIs, 34 % had participated in high-risk sports including rugby, soccer, cricket, lacrosse, judo and squash, and 18.8 % were military veterans [24]. However, it is unclear if all the cases with CTE changes described by Ling and colleagues would have met strict criteria for CTE using these newly defined NINDS guidelines. Furthermore, the relationship between non-diagnostic, non-specific astrocytic p-tau pathology and a history of traumatic exposure remains to be determined (Kovacs, in press).

**Fig. 6:**
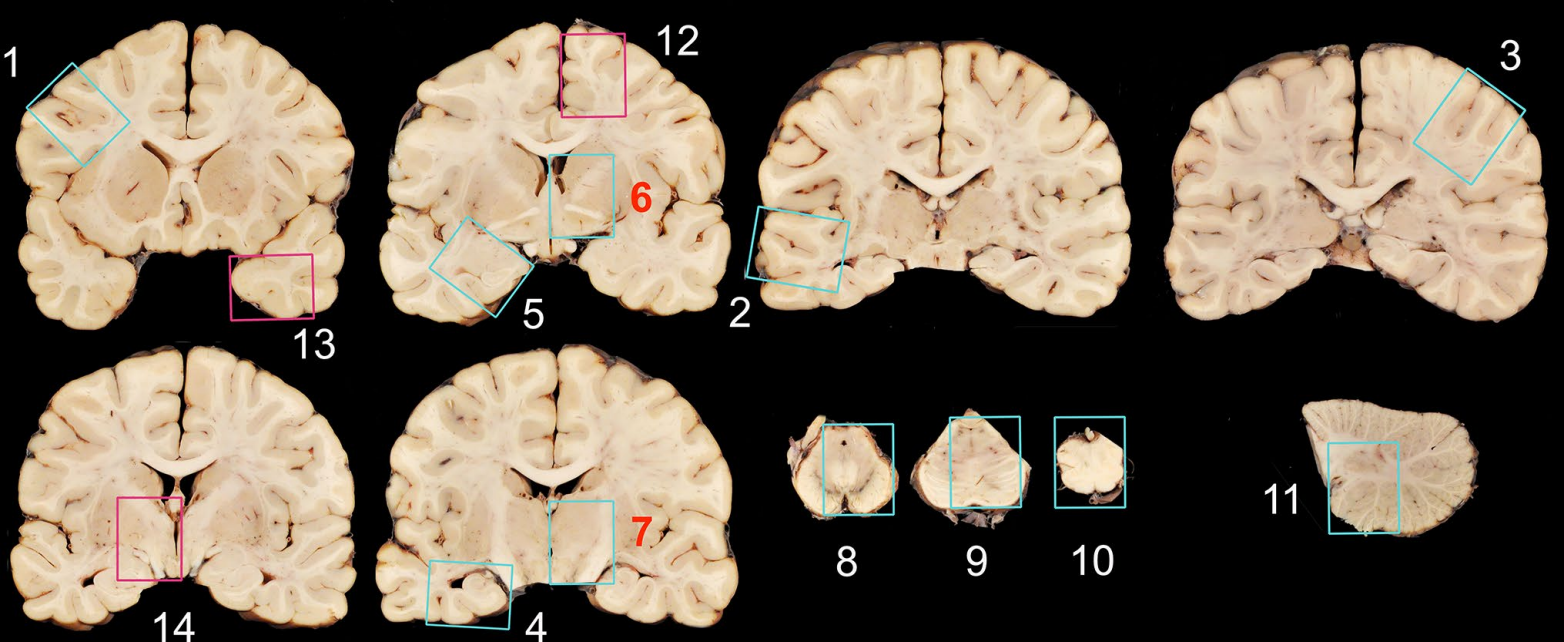
Minimum recommended brain regions for evaluation for CTE. The following sections from the NIA-AA blocking scheme are recommended for p-tau immunostaining in evaluation for CTE (*blue rectangles*). In the cortical sections (blocks *1*–*5*, *12*, *13*), the depths of the cortical sulci should be included in the section. *1* Middle frontal gyrus, *2* superior and middle temporal gyri, *3* inferior parietal lobule, *4* hippocampus, *5* amygdala and entorhinal cortex, *6* basal ganglia at level of anterior commissure with basal nucleus of Meynert, *7* thalamus, *8* midbrain with substantia nigra, *9* pons with locus coeruleus, *10* medulla oblongata, *11* cerebellar cortex and dentate nucleus; additional sections if high suspicion of CTE (*red rectangles*): *12* superior frontal gyrus, *13* temporal pole, *14* hypothalamus and mammillary body

At the present time, CTE remains a diagnosis that can only be made definitively upon neuropathological examination of the brain. Because the pathological diagnosis requires p-tau immunohistochemistry and the lesions are irregularly distributed, the detection of CTE in autopsy cohorts may require additional sampling compared to routine practices. The consensus panel’s minimum recommended sampling for CTE is found in Table 3. Sampling follows the protocol recommended by Alzheimer Disease Centers (National Institute on Aging-Alzheimer’s Association (NIA-AA) [34]) with the further recommendation that all cortical sections be taken to include the region at the depths of the cortical sulci. This has been shown in pilot studies to detect 80 % of CTE cases; however, 20 % of CTE cases, all early stage, would be missed by this sampling scheme [9]. Of the NIA-AA sampling guidelines, the following blocks are most valuable for detecting CTE: sulcal depths of the superior and middle frontal gyrus, superior and middle temporal gyrus and inferior parietal gyrus (Fig. 6). Of note, the Bielschowsky silver stain does not always detect the diagnostically significant focal perivascular cortical tau lesions, and the panel recommended p-tau immunohistochemistry for the diagnosis of CTE using AT8 immunostaining or equivalent p-tau antibody (CP-13 or PHF-1). The question of how extensive the sampling must be to “rule out” CTE was discussed, but no data were available to make this determination.

**Table 3:** Recommended brain regions to be sampled and evaluated

Region	CTE		
Middle frontal gyrus*	pTau^a^	pTDP-43	Aβ^c^
Superior and middle temporal gyri*	pTau^a^		
Inferior parietal lobule*	pTau^a^		Aβ^c^
Hippocampus and entorhinal cortex	pTau	pTDP-43^b^	Aβ^c^
Amygdala	pTau	pTDP-43^b^	
Thalamus	pTau		
Basal ganglia with basal nucleus of Meynert	pTau		
Midbrain including substantia nigra	pTau		
Pons including locus coeruleus	pTau		
Medulla including dorsal motor nucleus of vagus	pTau		
Cerebellar cortex and dentate nucleus	pTau		
Additional sections if high suspicion			
Superior frontal gyrus	pTau^d^		
Temporal pole	pTau^d^	pTDP-43	
Hypothalamus including mammillary body	pTau^d^		

In addition to the NIA-AA recommended regions for the evaluation of Alzheimer’s disease (AD) neuropathologic change and Lewy body disease (LBD) [34], we recommend wider p-tau screening to capture CTE and other tauopathies. In addition, if there is a high index of suspicion of CTE, we recommend taking extra sections of frontal and temporal cortices, and hypothalamus including the mammillary body

Bilateral representative sections from each region are recommended if both cerebral hemispheres are available for microscopic analysis

* Most valuable for detecting CTE neuropathology

^a^AT8 or equivalent Tau (CP-13 or PHF-1) on all cortical sections, if positive: stain other areas and possibly sample additional areas^d^. We do not recommend thioflavin or silver stains for the detection of CTE lesions

^b^TDP-43: amygdala and hippocampus, if positive then temporal pole and frontal cortex

^c^Aβ: middle frontal gyrus, inferior parietal lobule and hippocampus and entorhinal cortex; if positive wider sampling is recommended

^d^If there is a high index of suspicion consider taking extra sections, specifically superior frontal gyrus, temporal pole, and hypothalamus including mammillary body

These criteria are the beginning of the process to fully characterize the pathology of CTE, and this is only the first of a series of consensus conferences on the subject funded by the U01 NINDS research initiative. Many important questions were not addressed in this first consensus panel, including the degree of neuronal cell loss, gliosis, inflammation, and hemosiderin deposition, and the diagnosis of CTE in the presence of comorbid pathologies, including AD. Future directions will include further validation of the neuropathological criteria for CTE, including staging of the severity of p-tau pathology and characterization of early disease. More pathological characterization will also be necessary to delineate the involvement of the other subcortical regions, including amygdala, globus pallidus, subthalamic nucleus, accumbens, neostriatum, thalamus, midbrain, cerebellum, spinal cord and white matter. It will also be important to determine the differential hippocampal p-tau pathology in CTE compared to AD, whether the TDP-43 pathology is distinctive for CTE and the contribution of hippocampal sclerosis and TDP-43 deposition to the clinical and pathological features. Population isolates that develop unusual p-tau pathologies will need to be distinguished from CTE pathology in addition to Guam, such as the Kii peninsula of Japan [23]. In addition, the contributions of other proteinopathies, including β-amyloidosis (diffuse and dense core Aβ plaques and amyloid angiopathy) and alpha-synuclein will be important to determine. Similarly, the role of microvascular pathology, iron deposition, axonal injury, neuroinflammation and astrocytosis to the pathogenesis of CTE pathology needs resolution.

Future investigation will be needed to understand the relationship of the pathology to the clinical symptoms, genetics, neuroimaging and other biomarkers (including p-tau positron emission tomography (PET) imaging and cerebrospinal fluid (CSF) and blood biomarkers), metabolomics, proteomics, and epigenetics. It will also be important to determine whether specific “tau strains” are involved in the development of CTE. Furthermore, more information is needed regarding the frequency, severity, and nature of the traumatic exposures, length of survival after trauma, as well as factors such as the age at first and last exposure to trauma, and the effects of military compared to civilian brain trauma.

The limitations of the present study include the relatively small sample set, the use of digitized images, the selection of suspected CTE cases by a single source, the use of representative cases of moderate-to-late stage severity of CTE, and presence of some age-related co-morbidities. However, these limitations are offset by the fact that all evaluating neuropathologists were evaluating the exact same digital images, the cases were all uniformly prepared by a central laboratory, and the evaluation was performed blinded to all clinical or demographical data and gross neuropathological findings. Other limitations to the present study include the lack of data regarding TBI history in the non-CTE cases under evaluation. Future studies are being designed to specifically address the contribution of TBI at all levels of severity to neurodegenerative pathologies.

## Conclusion

A consensus panel of 7 neuropathologists blinded to all clinical conditions and demographics evaluated the identical digitized images of 25 cases representing various tauopathies and concluded that the pathology of CTE is distinct from other tauopathies. In addition, the panel described the pathognomonic lesion of CTE as an accumulation of abnormal tau in neurons and astroglia distributed perivascularly at the depths of sulci in the isocortex in an irregular pattern. Future consensus meetings will address validation of the criteria among a wider group of neuropathologists using cases submitted from multiple sources. In addition, future meetings will address the identification of comorbid CTE when other neurodegenerative diseases and other diseases are present. Furthermore, additional research will be necessary to determine the contribution of p-tau and other pathologies to the development of clinical symptoms of CTE.

The incidence and prevalence of CTE remain unknown and will likely require methods of in vivo detection and diagnosis to make a clear determination. This first consensus conference on the pathological criteria for CTE represents the first step along the path to standardizing the neuropathology of CTE and paving the way for future determinations of specific clinical symptomatology and refinements in clinical diagnosis.

### Electronic supplementary material

Supplementary material 1 (DOCX 8564 kb)

Supplementary material 2 (DOCX 113 kb)

Supplementary material 3 (DOCX 152 kb)

## Appendix: TBI/CTE group

Elissa Flannery, Daniel H. Daneshvar, Patrick T. Kiernan, Jesse Mez, Lauren Murphy, Todd M. Solomon, Debra Babcock, Patrick S. F. Bellgowan, and Walter J. Koroshetz.

## Footnotes

1 Although often referred to as a *soccer* player, the young subject with CTE described by Geddes et al. 1999 as the “keen amateur footballer who frequently “headed” the ball while playing” [13], was an amateur *rugby* player (personal communication, T. Revesz).

2 Neuropathologist present at the face-to-face panel discussion, but did not participate in the slide evaluations.
