# Supplementary material for: The first NINDS/NIBIB consensus meeting to define neuropathological criteria for the diagnosis of chronic traumatic encephalopathy
Source: Acta Neuropathol. 2015 Dec 14;131:75–86. doi: 10.1007/s00401-015-1515-z (PMC4698281; doi:10.1007/s00401-015-1515-z)
Supplement: Supplementary file 1 — Supplementary material 1 (DOCX 8564 kb) [file 401_2015_1515_MOESM1_ESM.docx]

# Neuropathological Criteria for Tauopathies

# 1. Chronic Traumatic Encephalopathy

Repetitive brain trauma is associated with a progressive neurological deterioration, originally termed dementia pugilistica, and more recently, chronic traumatic encephalopathy (CTE). Most instances of CTE occur in association with the play of collision sports, such as American football and boxing, but CTE has also been reported blast injuries from improvised explosive devices, physical abuse, poorly controlled epilepsy, and head-banging behaviors (Corsellis et al., 1973; Geddes et al., 1999; McKee et al., 2009, 2013; Omalu et al., 2005). Like many other neurodegenerative diseases, CTE is diagnosed with certainty only by neuropathological examination of brain tissue.

The most commonly encountered macroscopic changes in brains affected by CTE include reduced brain weight; cerebral atrophy, that is typically most severe in the frontal and anterior temporal lobes, enlargement of the lateral and third ventricles; cavum septum pellucidum; septal fenestrations; atrophy of the diencephalon and mammillary bodies and pallor of the locus coeruleus and substantia nigra. Grossly identifiable changes are rarely found in the early stages of CTE and are more characteristic of advanced disease. Although cerebellar abnormalities were described in the initial reports of CTE affecting boxers, grossly identifiable cerebellar abnormalities are rarely present in CTE associated with other sports or activities (McKee et al., 2013). Symptoms of CTE include behavioral and mood changes that may begin in early middle age. Short-term memory loss, executive dysfunction, cognitive impairment and dementia are common symptoms in later life with more advanced disease.

CTE is a tauopathy and is characterized by the deposition of hyperphosphorylated tau (p-tau) protein as neurofibrillary tangles (NFTs), astrocytic tangles (ATs) and neurites in the neocortex and medial temporal lobe. The NFTs in CTE often show a perivascular distribution and an irregular clustering at the depths of the sulci. NFTs also preferentially involve of the superficial cortical layers, a feature that is most prominent in temporal isocortex. The frontal, temporal, septal, insular and parietal cortices are primarily affected, while primary visual and occipital cortices are generally spared. In advanced disease, the medial temporal lobe structures show pronounced neuronal loss and gliosis, with a high density of NFTs, including extracellular ghost tangles. In approximately 80% of cases, there are also TDP-43 immunoreactive neurites and intraneuronal inclusions. The following criteria for the neuropathological diagnosis of CTE are proposed (McKee et al., 2013):

1. **Perivascular foci of p-tau immunoreactive neurofibrillary tangles (NFTs) and astrocytic tangles (ATs) in the neocortex**
2. **Irregular distribution of p-tau immunoreactive NFTs and ATs at the depths of cerebral sulci**
3. **NFTs in the cerebral cortex located preferentially in the superficial layers (often most pronounced in temporal cortex)**
4. **Supportive features: Clusters of subpial ATs in the cerebral cortex, most pronounced at the sulcal depths.**


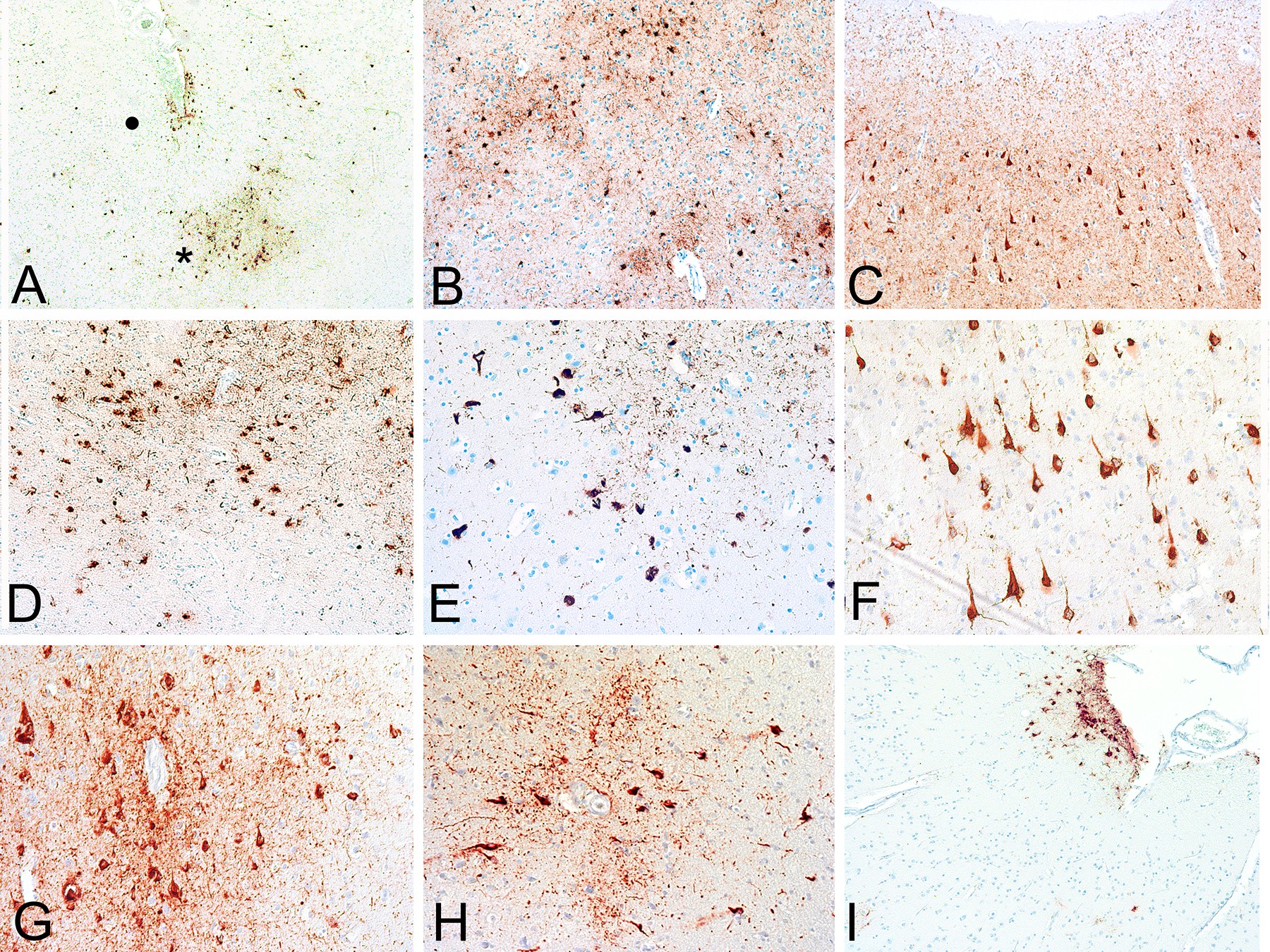


# Figure 1. Microscopic CTE p-tau pathology

**A.** Clusters of NFTs and glial tau pathology are often found at the depths of the sulci in the frontal, insular and temporal cortices (*) often associated with clusters of subpial ATs (•).

1. **. D., E., G., H.** There is often an accentuation of the NFTs around small blood vessels
2. **. F**. The NFTs preferentially involve the superficial layers of cortex, a feature that is often most prominent in the temporal lobe.

**I.** Clusters of subpial ATs are also common in CTE.

# Alzheimer’s Disease (AD)

Extracellular deposits of ß-amyloid (Aß) peptides, or senile plaques, and NFTs are considered essential neuropathological features of AD. The recent National Institute on Aging-Alzheimer’s Association guidelines for the neuropathological assessment of AD include an “ABC” score that incorporates histopathological assessments of amyloid ß deposits (A), staging of NFTs (B), and scoring of neuritic plaques (C) (Montine et al, 2012) however, for the purposes of this consensus conference, such detailed analysis is not necessary.


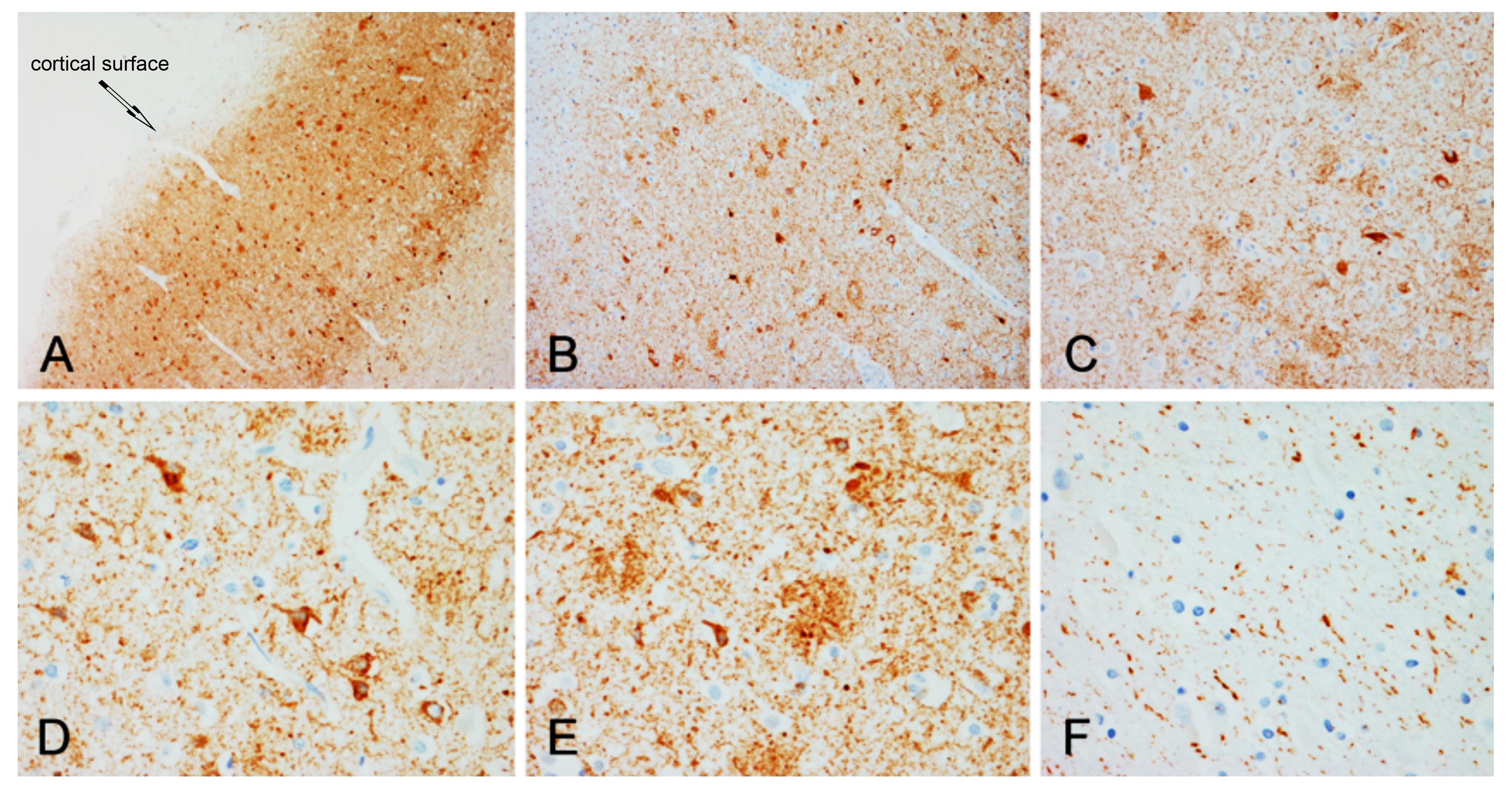


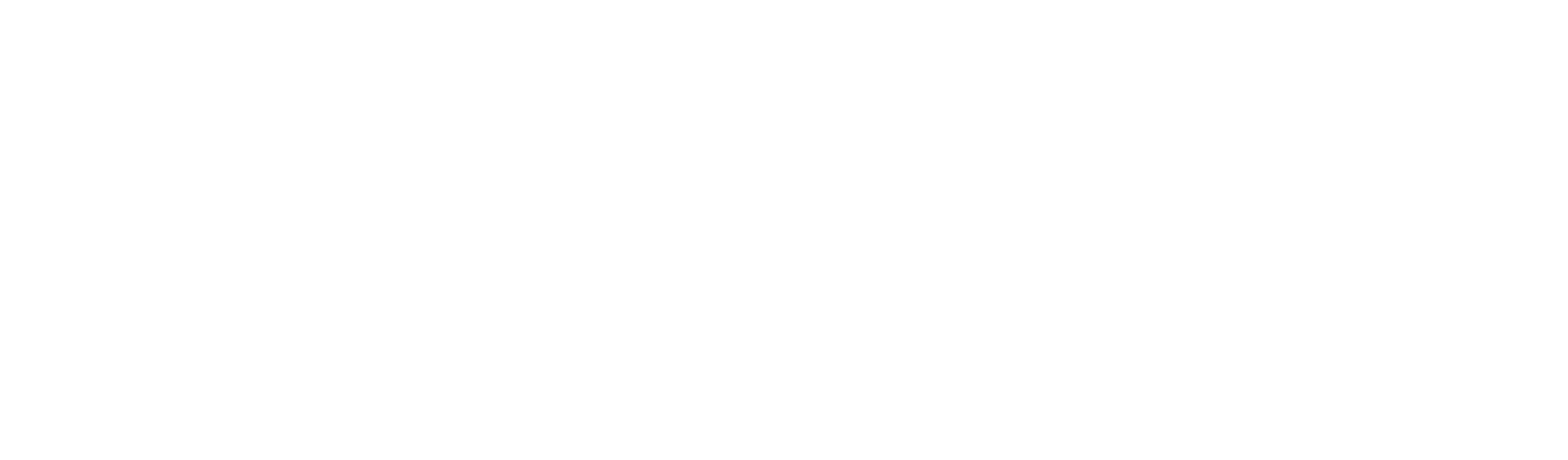


**Figure 2. Microscopic AD p-tau pathology**

**A.** Dense tau pathology is found throughout all cortical layers, with laminar accentuation in layers III and

V. There is no preferential distribution around penetrating vessels or at the pial surface. Lamina I often has a paucity of tau. **B. & C.** Phospho-­‐tau immunohistochemistry reveals a mixture of neurofibrillary tangles (NFT), neuritic plaques (NP) and neuropil threads. **D.** Pretangles and NFT occur in affected cortices. (Note: lack of association of tau pathology with blood vessel) **E.** Neuritic plaques are characteristic. **F.** There is little tau pathology in the white matter, when it occurs it consists primarily of threads immediately beneath cortical ribbon.

**3. Primary Age-­‐related Tauopathy (PART) also known as Neurofibrillary Predominant Senile Dementia**

Most patients with Primary Age-related Tauopathy are found to have mild‐to-moderate AD-type neurofibrillary degeneration in the medial temporal lobe, but lack Aß plaques (Crary et al., 2014). PART is generally considered is a disorder of aging and is commonly clinically associated with an amnestic syndrome (Dickson, 2009).

Neuropathologically, PART is characterized by diffuse cerebral atrophy with the most severe atrophy in the medial temporal lobe. NFTs are found primarily in the hippocampus, amygdala and medial temporal cortex with fewer in the cerebral cortex. Many of the NFTs are extracellular “ghost” tangles. There may be diffuse amyloid deposits; but there are usually no or very few neocortical neuritic plaques (Dickson, 2009).


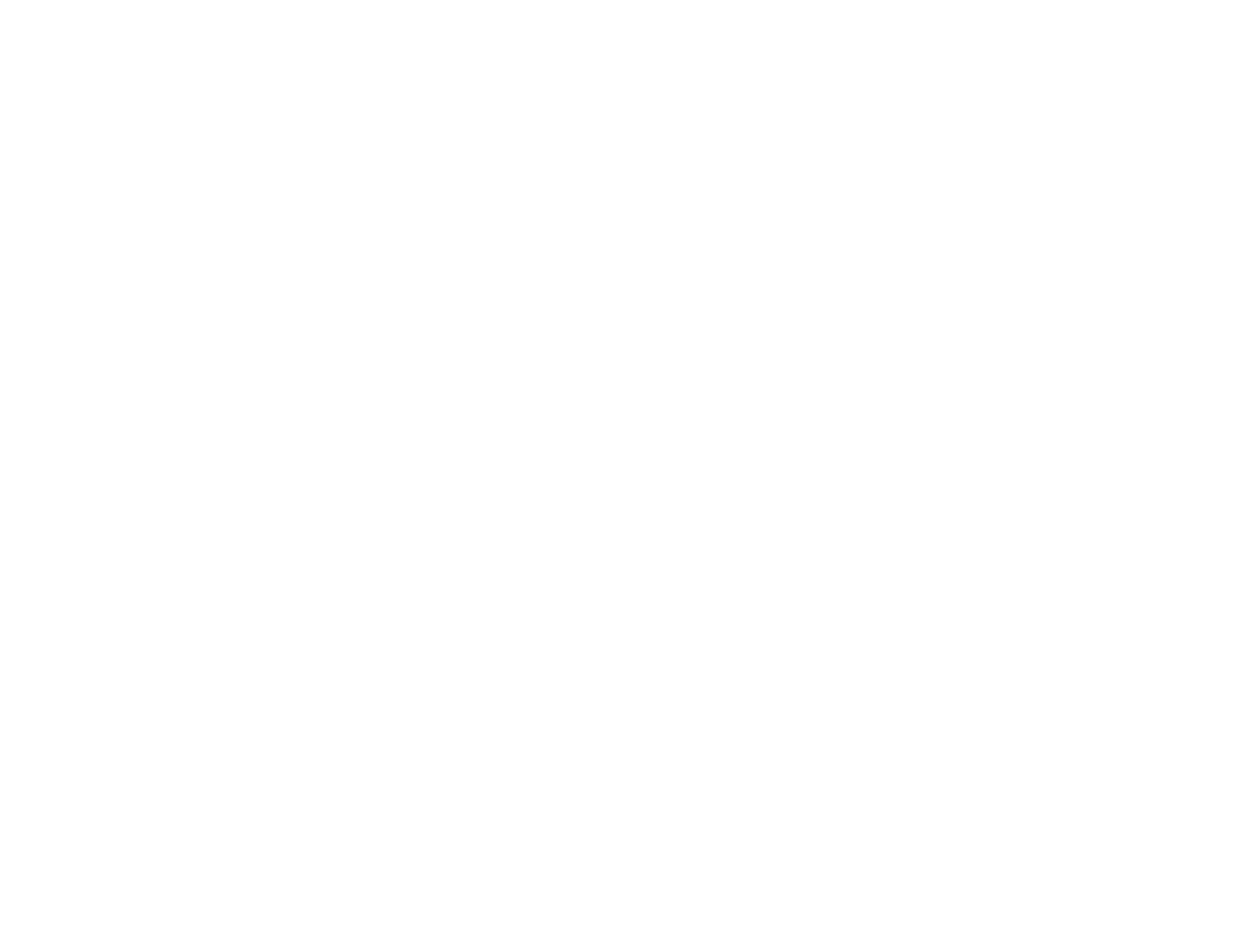

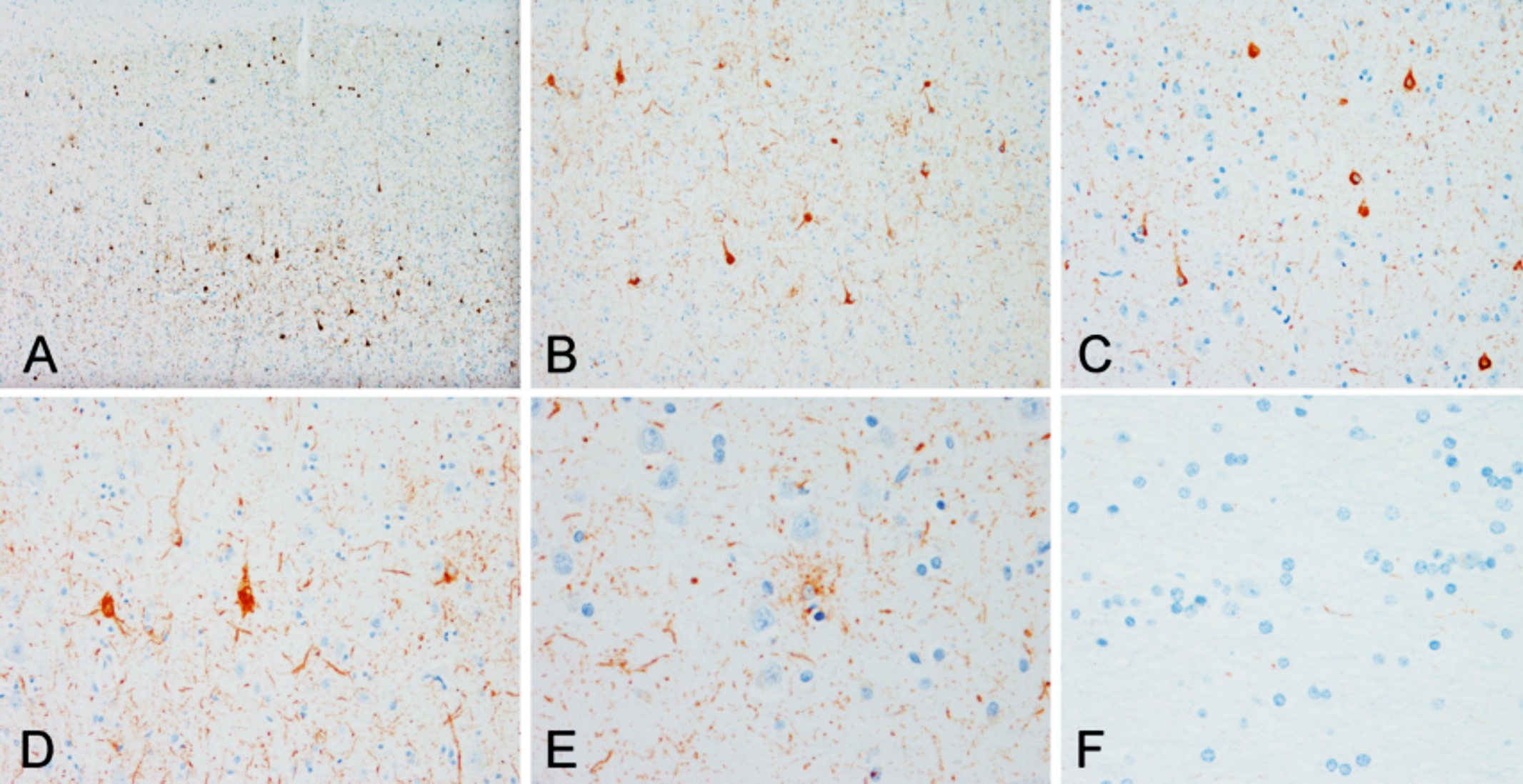


**Figure 3. Microscopic PART (NFT dementia) p-­‐tau pathology**

**A.** Tangle predominant pathology in outer and inner pyramidal cell layers of the entorhinal cortex. There is no preferential distribution around penetrating vessels or at the pial surface. Lamina I has a paucity of tau. **B. & C.** Phospho-­‐tau immunohistochemistry reveals neurofibrillary tangles (NFT) and variable neuropil threads. **D.** Pretangles and NFT are found in affected cortices. **E.** Tau accumulation in cortical astrocytes is uncommon. **F.** There is little white matter tau pathology, when it is present; it consists of a few threads.

# Parkinson dementia complex of Guam (GPDC)

Guamanian PDC is an endemic disease affecting the Chamorro people of Guam characterized by progressive dementia and parkinsonism (Dickson, 2009).

Guam PDC is pathologically characterized by diffuse cerebral atrophy that is accentuated in the frontal and temporal lobes. There is often also atrophy of the hippocampus and parahippocampal gyrus, basal ganglia, thalamus and brainstem. The substantia nigra and locus coeruleus show loss of pigmentation. Microscopically the frontal, insular and temporal cortices show neuronal loss with NFTs distributed primarily in the superficial cortical layers. Most cases have few senile plaques. The cerebral white matter shows severe atrophy but myelin pallor and tau-positive thread-like structures are not present in most cases. In areas of severe pathology (e.g. hippocampus), many of the NFTs are extracellular. Tau positive glial inclusions and coiled oligodendroglial inclusions may be present. (Oyanagi K et al, 2011).


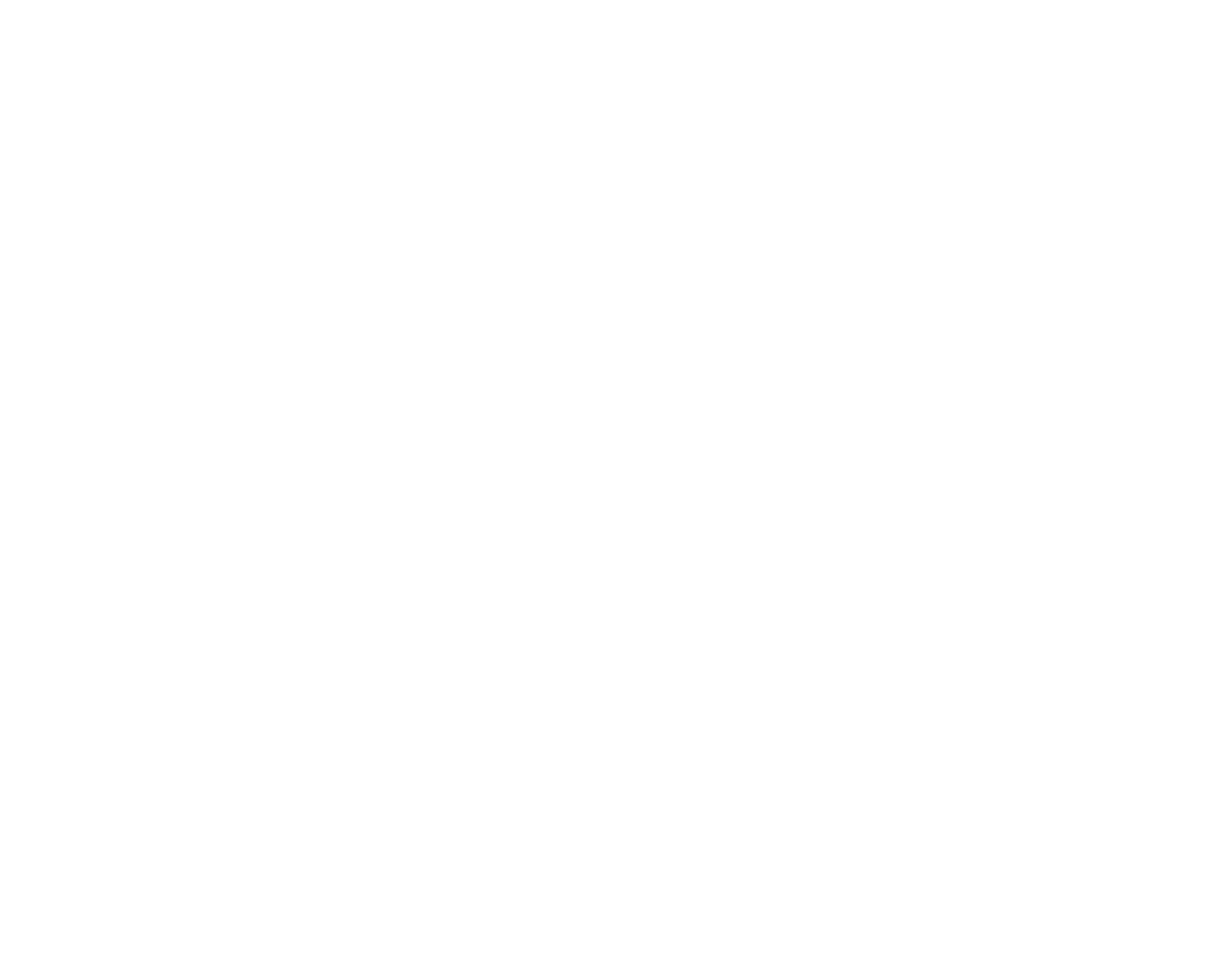

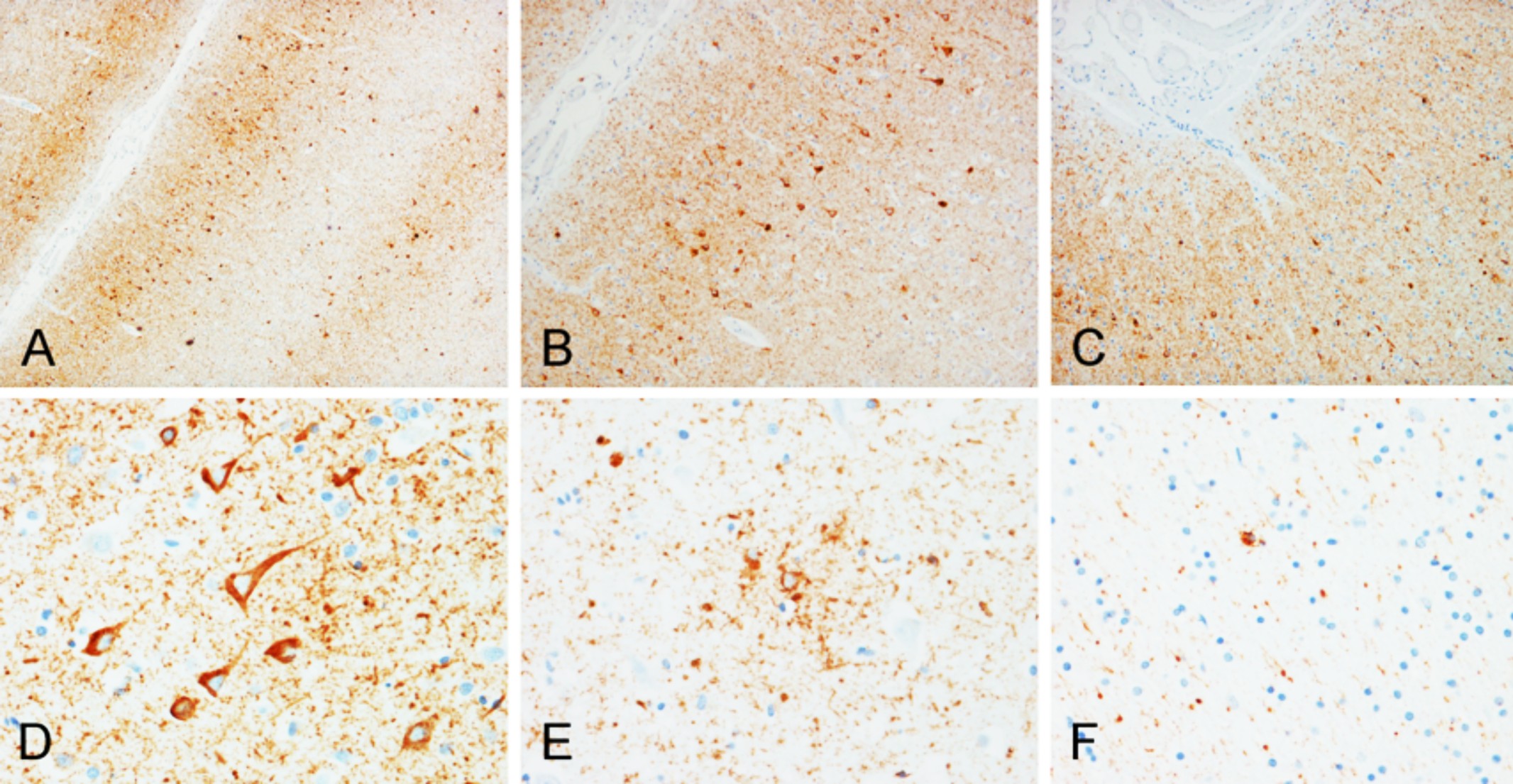


**Figure 4. Microscopic Guam PDC p-tau pathology**

**A.** Tangle predominant pathology is found in the outer and inner pyramidal cell layers of the entorhinal cortex, similar to PART, but it is usually more dense. There is no preferential distribution around penetrating vessels or at the pial surface. Lamina I has a paucity of tau. **B.** Phospho-tau immunohistochemistry reveals NFT and many neuropil threads. **C.** No preferential tau pathology is found at the depth of sulcus or in the perivascular regions. **D.** The affected cortices show mostly mature NFT (Note: p-tau does not detect extracellular NFT). **E.** Tau accumulation occurs in “granular hazy” astrocytes. **F.** There is little white matter tau pathology, when present; it consists of a few threads and a few oligodendroglial-coiled bodies.

# Argyrophilic grain disease (AGD)

AGD is detected in about 5% of cases of late-onset dementia; it is common in mild cognitive impairment and may be co-exist with other neurodegenerations, including AD, CBD and PSP.

Pathologically, comma-shaped or grain-like structures are found in the neuropil of the medial temporal lobe, and in rare cases more widely distributed in limbic and frontotemporal distribution (“Diffuse AGD”). There is neuronal tau pathology in the form of pretangles, as well as positive oligodendroglia “coiled bodies” and ramified astrocytes. Ballooned neurons are also very common (Tolnay et al., 2004, Cairns et al., 2007).


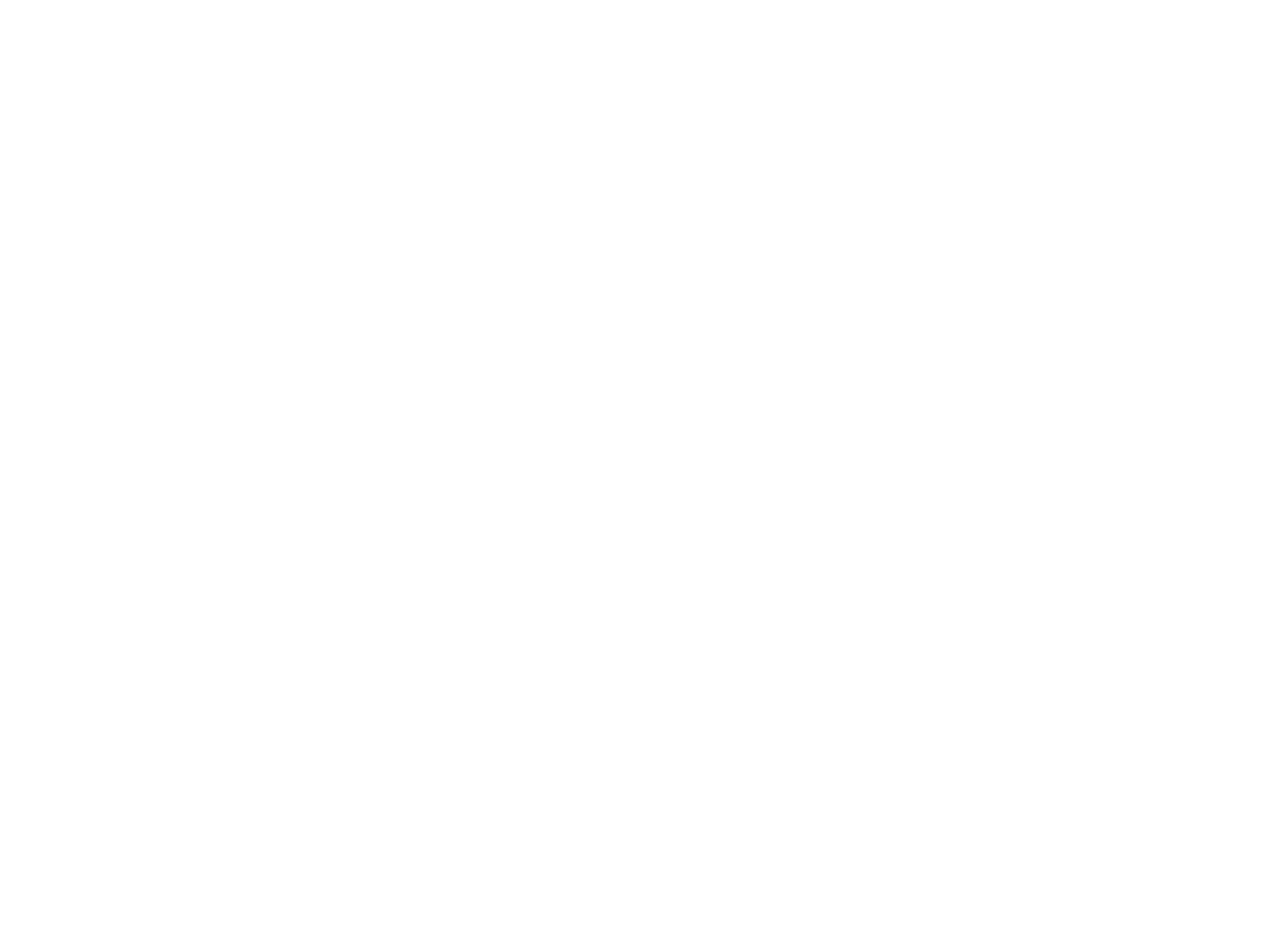

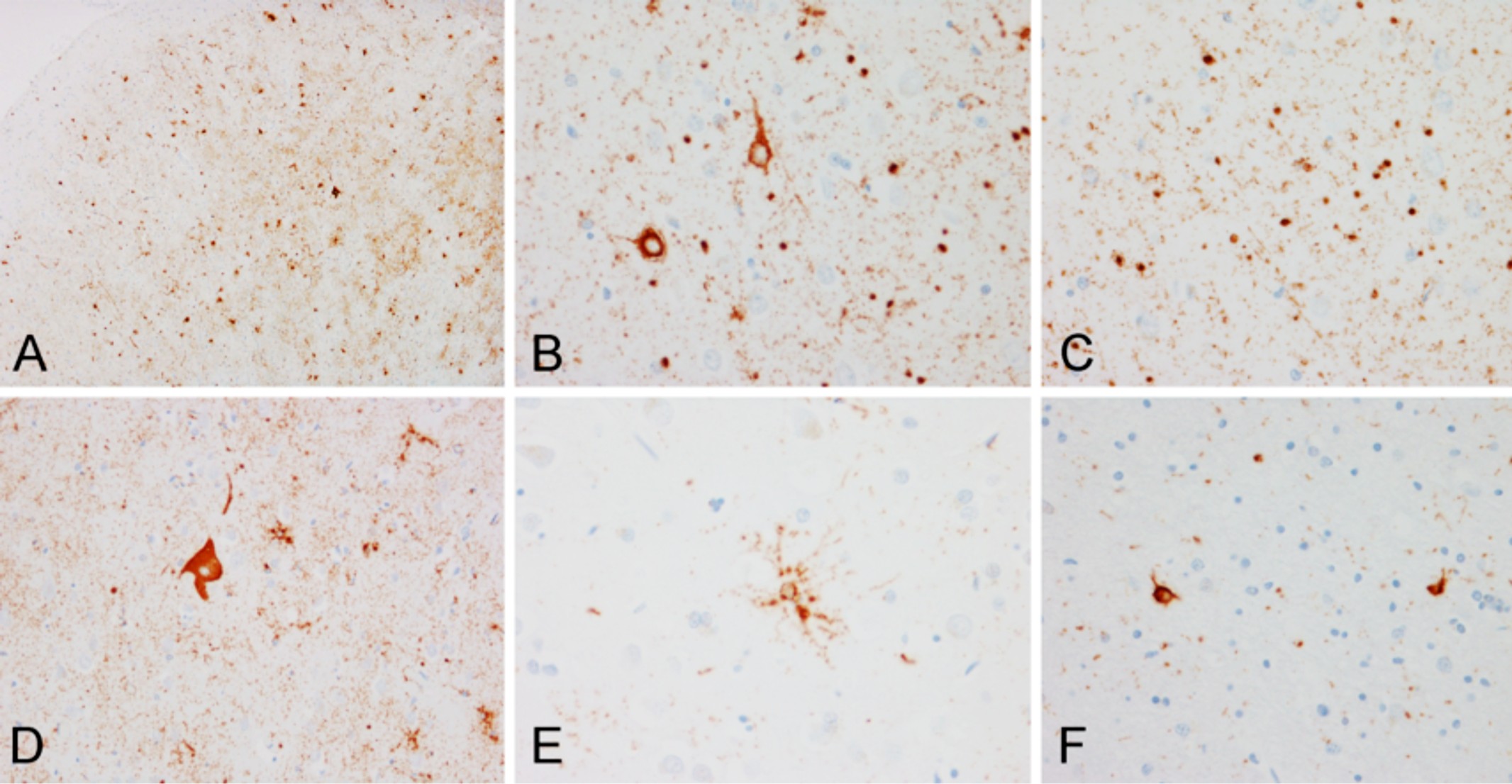


**Figure 5. Microscopic AGD p-tau pathology**

**A.** Immunohistochemistry for p-tau shows grains in the upper cortical layers associated with diffuse neuropil immunoreactivity. B. Neuronal perikaryal lesions consist primarily of pretangles. C. Grains display dense spherical profiles about 2-3-μm in diameter and are associated with variable numbers of neuropil threads. D. A ballooned neuron in the lower cortical layer shows diffuse p-­‐tau cytoplasmic staining. There are also a few ramified astrocytes with diffuse staining of the neuropil. E. P-­‐tau-­‐immunopositive astrocytes with ramified granular cell processes may be present in neocortex. F. White matter tau pathology is sparse and includes oligodendroglial-coiled bodies.

# Corticobasal degeneration (CBD)

CBD is associated with various clinical presentations including movement disorders (akinesia, rigidity, limb dystonia, focal reflex myoclonus, postural/action tremor, postural instability); cerebral cortical features (cortical sensory loss, apraxia, alien limb, frontal release signs, dementia, dysphasia); and other features (corticospinal tract signs, oculomotor dysfunction, eyelid motor dysfunction, dysarthria, dysphagia).

Macroscopically, there may be focal cortical degeneration of the superior frontal gyrus and superior parietal lobule, or focal atrophy of the frontal lobes or language areas. There is also nigral pallor.

Microscopically, there are many tau positive thread-­‐like processes in the gray and white matter, as well as tau accumulation in neurons and astrocytes. The most specific lesion is the astrocytic plaque. There are usually swollen cortical neurons or ballooned neurons. (Kouri N et al., 2011, Cairns et al., 2007).


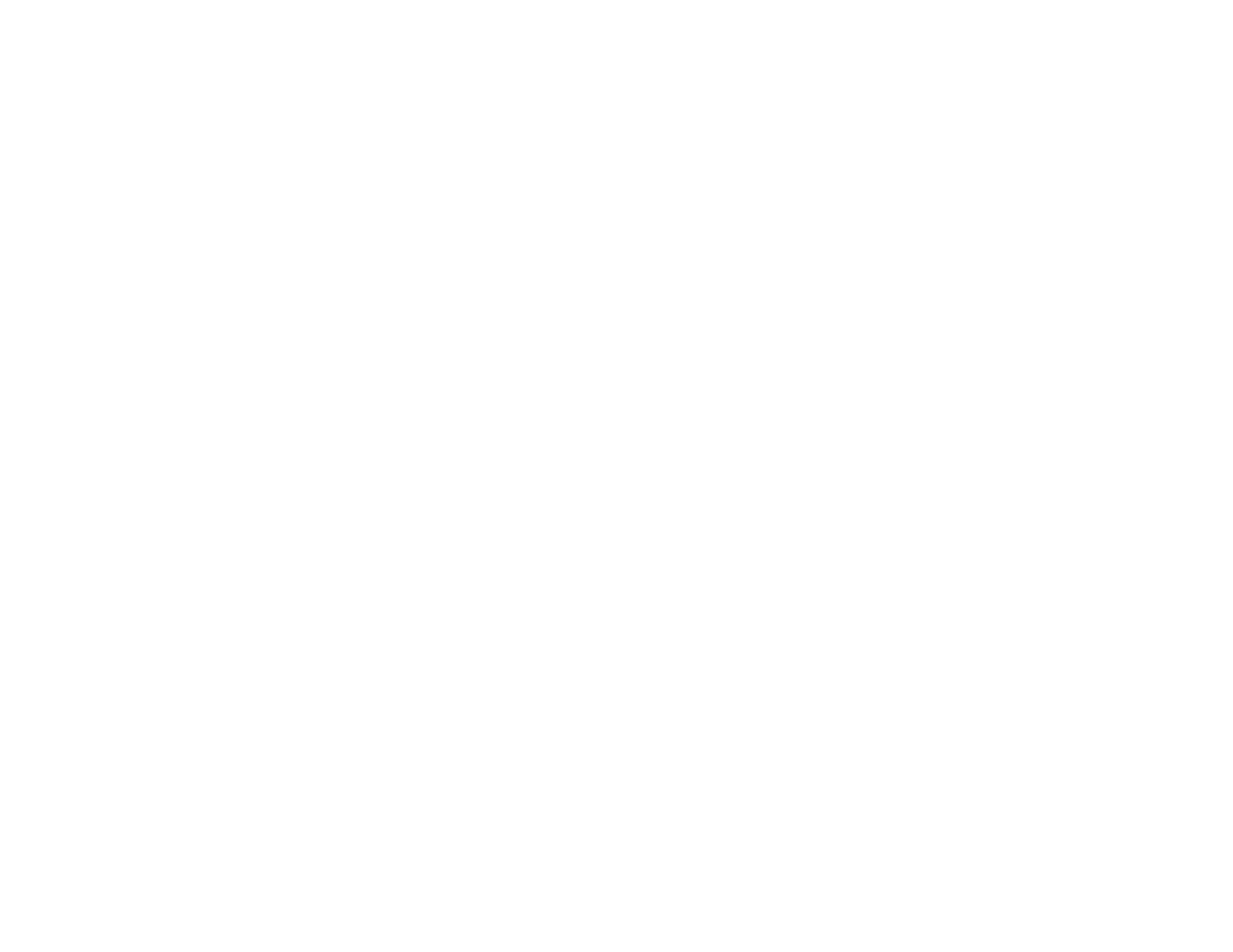

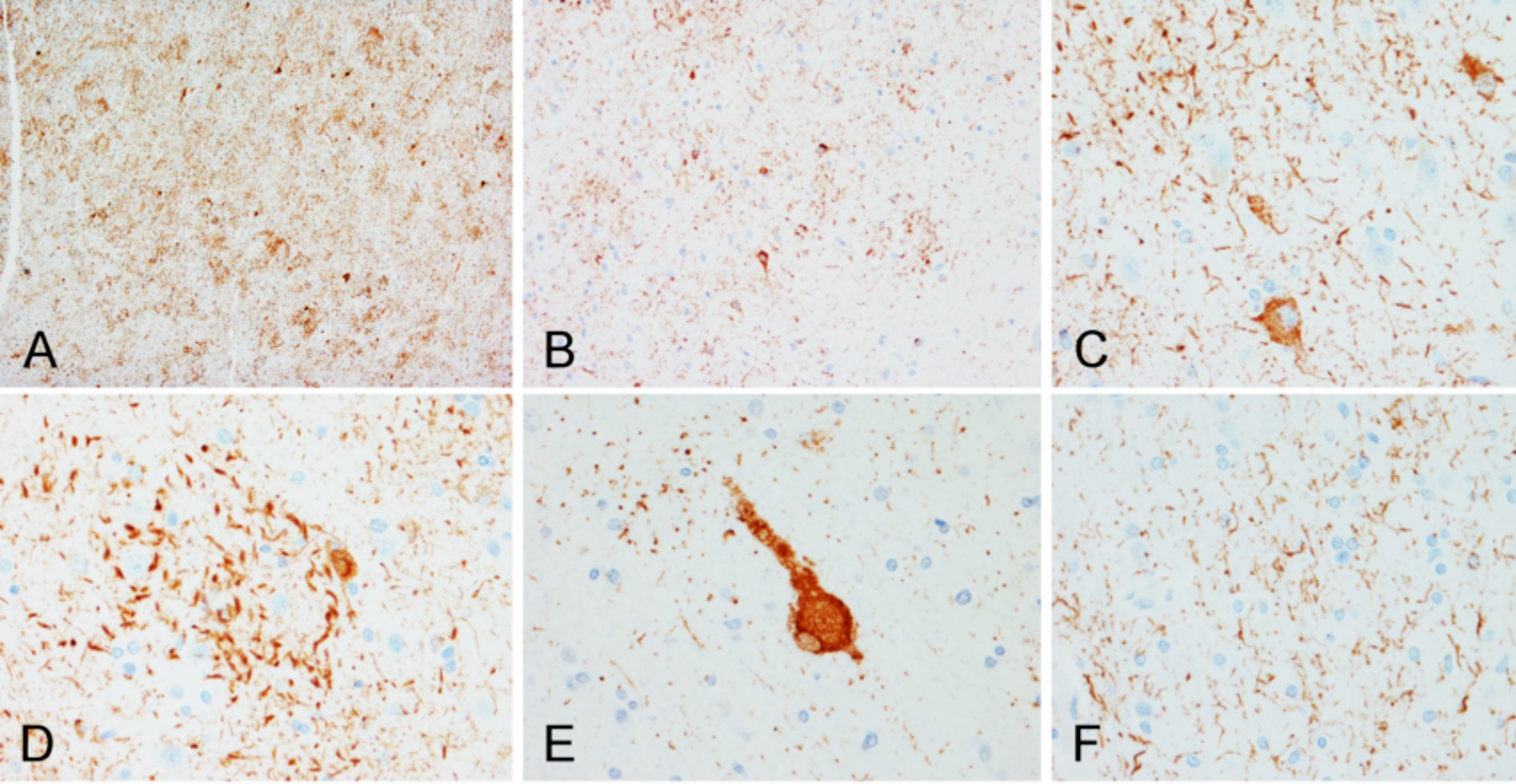


**Figure 6. Microscopic CBD p‐tau pathology**

**A.** In CBD, the cortical gray matter has extensive tau pathology that does not show an obvious laminar distribution and blends imperceptibly into the white matter. **B.** Phospho-­‐tau immunohistochemistry reveals plaque like lesions, neurons and neuropil threads**. C.** Many neuropil threads and neurons with pretangles or pleomorphic cytoplasmic inclusions may be found. **D.** Astrocytic plaques consist of clusters of short, stubby, sometimes fuzzy, cell processes around a central astrocyte that is usually unstained. **E.** Ballooned neurons have diffuse cytoplasmic swelling and variable p-­‐tau immunoreactivity. **F.** White matter tau pathology is often abundant and characterized by many threads and a few coiled bodies (not shown).

# Progressive supranuclear palsy

Progressive supranuclear palsy (PSP; Steele‐Richardson-Olszewski syndrome) is a tauopathy with predominant tau pathology and prominent Parkinsonism. Clinical signs at onset are most frequently impaired mobility, falls, cognitive deficits, and bulbar signs.

The neuropathologic features of PSP include predominant midbrain atrophy; to a lesser extent, atrophy of the pallidum, thalamus, and subthalamic nucleus; and mild symmetric frontal atrophy. The histopathology is characterized by neuronal changes (globose and flame‐shaped neurofibrillary tangles, neuropil threads) and glial tau pathology (tufted astrocytes, thorn-shaped astrocytes, oligodendroglial coiled bodies), that show a distinct topographic distribution (Litvan I et al., 1996, Cairns et al., 2007).


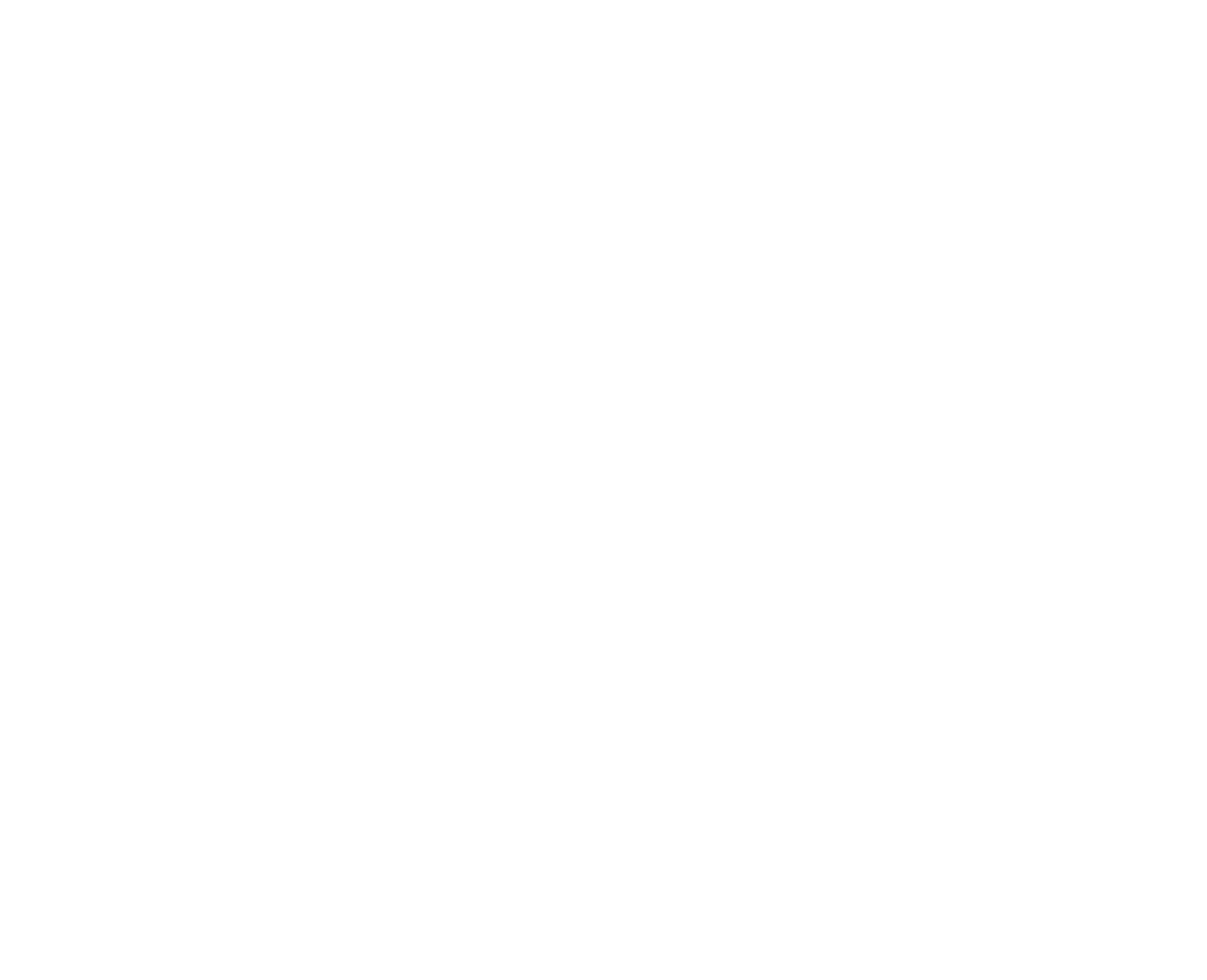

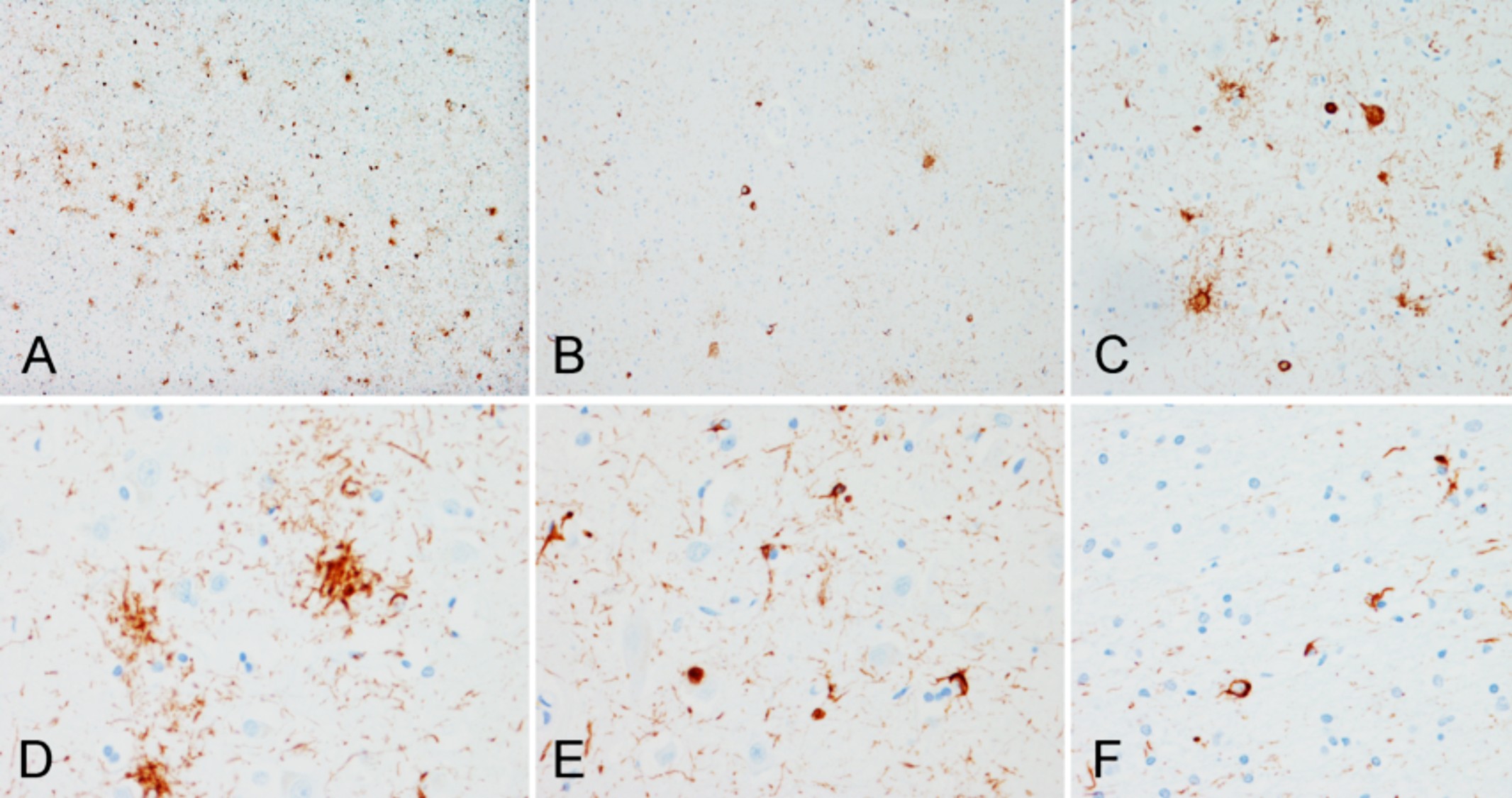


**Figure 7. Microscopic PSP p-­‐tau pathology**

**A. and B.** In PSP, cortical gray matter has variable tau pathology that is most dense in primary motor cortex (**A.**) and less dense in frontal association cortices (**B.**). Tau positive lesions do not show an obvious laminar distribution and are not specifically perivascular or subpial. **C.** Phospho-­‐tau immunohistochemistry reveals both neuronal and glial lesions, as well as neuropil threads**. D.** Tufts of abnormal fibers, or “tufted astrocytes” are the hallmark lesion of PSP and are characterized by tau in the cell body and proximal cell processes. **E. and F.** Oligodendroglial coiled bodies are found in both cortical gray matter (**E.**) and white matter (**F.**). Threads are usually more abundant in cortical gray matter.

Bibliography

Cairns NJ, Bigio EH, Mackenzie IR, Neumann M, Lee VM-Y, Hatanpaa KJ et al. (2007) Neuropathologic diagnostic and nosologic criteria for frontotemporal lobar degeneration: consensus of the Consortium for Frontotemporal Lobar Degeneration. Acta neuropathologica 114:5-22

Corsellis J, Bruton C, Freeman-­‐Browne D. 1973. The aftermath of boxing. *Psychol. Med.* 3: 270-­‐303

Crary JF, Trojanowski JQ, Schneider JA, Abisambra JF, Abner EL, et al. 2014. Primary age-­‐related tauopathy (PART): a common pathology associated with human aging. *Acta Neuropathol*. 2014 Dec;128(6):755-66.

Dickson DW. 2009. Neuropathology of non-­‐Alzheimer degenerative disorders. *Int. J. Clin. Exp. Pathol.* 3: 1-­‐23

Geddes J, Vowles G, Nicoll J, Revesz T. 1999. Neuronal cytoskeletal changes are an early consequence of repetitive head injury. *Acta Neuropathol.* 98: 171-­‐78

Kouri N, Whitwell JL, Josephs KA, Rademakers R, Dickson DW. 2011. Corticobasal degeneration: a pathologically distinct 4R tauopathy. *Nature Reviews Neurology* 7: 263-­‐72

Litvan I, Hauw J, Bartko J, Lantos P, Daniel S, et al. 1996. Validity and reliability of the preliminary NINDS neuropathologic criteria for progressive supranuclear palsy and related disorders. *J. Neuropathol. Exp. Neurol.* 55: 97-­‐105

McKee AC, Cantu RC, Nowinski CJ, Hedley-­‐Whyte ET, Gavett BE, et al. 2009. Chronic traumatic encephalopathy in athletes: progressive tauopathy after repetitive head injury. *J. Neuropathol. Exp. Neurol.* 68: 709-­‐35

McKee AC, Stern RA, Nowinski CJ, Stein TD, Alvarez VE, et al. 2013. The spectrum of disease in chronic traumatic encephalopathy. *Brain* 136: 43-­‐64

Montine TJ, Phelps CH, Beach TG, Bigio EH, Cairns NJ, et al. 2012. National Institute on Aging–Alzheimer’s Association guidelines for the neuropathologic assessment of Alzheimer’s disease: a practical approach. *Acta Neuropathol.* 123: 1-­‐11

Omalu BI, DeKosky ST, Minster RL, Kamboh MI, Hamilton RL, Wecht CH. 2005. Chronic traumatic encephalopathy in a National Football League player. *Neurosurgery* 57: 128-­‐34

Oyanagi K, Hashimoto T, Yamazaki M. 2011. Parkinsonism-­‐Dementia Complex of Guam in Neurodegeneration*. The molecular pathology of dementia and movement disorders.* Dickson D, Weller RO, eds. Wiley

Tolnay M, Clavaguera F. 2004. Argyrophilic grain disease: a late-­‐onset dementia with distinctive features among tauopathies. *Neuropathology* 24: 269-­‐83
