# Supplementary material for: The first NINDS/NIBIB consensus meeting to define neuropathological criteria for the diagnosis of chronic traumatic encephalopathy
Source: Acta Neuropathol. 2015 Dec 14;131:75–86. doi: 10.1007/s00401-015-1515-z (PMC4698281; doi:10.1007/s00401-015-1515-z)
Supplement: Supplementary file 2 — Supplementary material 2 (DOCX 113 kb) [file 401_2015_1515_MOESM2_ESM.docx]

**Table 1.** **Reviewer’s evaluation of presumptive CTE cases**

| **Age** | **Sex** | **Sport** | **SDX** | **COM** | **Reviewers Responses** | | | | | | |
| --- | --- | --- | --- | --- | --- | --- | --- | --- | --- | --- | --- |
| 60-65 | M | PBX | CTE III |  | CTE | CTE | CTE | CTE | CTE | CTE | CTE HS |
| 66-70 | M | PFB | CTE III |  | CTE | CTE | CTE | CTE | CTE | CTE HS | CTE PART AGD |
| 66-70 | M | PFB | CTE IV |  | CTE | CTE | CTE | CTE | CTE | CTE PART | CTE ADC HS |
| 76-80 | M | PFB | CTE IV | Aß | CTE ADC | CTE ADC | CTE ADC | CTE ADC | CTE AD | CTE AD | CTE AD |
| 60-65 | M | PFB | CTE IV | Aß | CTE | CTE | CTE ADC | CTE ADC | CTE ADC | CTE ADC | CTE AD |
| 66-70 | M | PFB | CTE IV | Aß | CTE | CTE | CTE ADC | CTE ADC | CTE ADC | CTE AD | CTE AD  HS |
| 66-70 | M | PFB | CTE IV | Aß | CTE | CTE | CTE | CTE HS | CTE AGD | CTE AD | AGD |
| 80-85 | M | PFB | CTE IV | Aß | CTE | CTE ADC | CTE ADC | CTE ADC | CTE AD HS | CTE AD HS | MSA |
| 66-70 | M | PFB | CTE IV | Aß | CTE | CTE | CTE | CTE ADC HS | CTE AD HS | HS | GPDC |
| 71-75 | M | PFB | CTE IV | Aß,  LBD | CTE ADC | CTE ADC | CTE AD HS | CTE AD HS | PSP | PSP | GPDC |
| Abbreviations: Aß Beta-amyloid plaques, AD Alzheimer’s disease, ADC Changes of Alzheimer’s disease, AGD Argyrophilic grain disease, COM Co-morbidities; GPDC Guamanian Parkinson’s Dementia Complex, HS Hippocampal Sclerosis, LBD Lewy body disease, MSA Multiple System Atrophy, PBX Professional Boxing, PFB Professional Football, SDX Submitted Diagnosis  Red text indicates discrepancies with submission diagnosis of CTE. | | | | | | | | | | | |
