# Supplementary material for: The first NINDS/NIBIB consensus meeting to define neuropathological criteria for the diagnosis of chronic traumatic encephalopathy
Source: Acta Neuropathol. 2015 Dec 14;131:75–86. doi: 10.1007/s00401-015-1515-z (PMC4698281; doi:10.1007/s00401-015-1515-z)
Supplement: Supplementary file 3 — Supplementary material 3 (DOCX 152 kb) [file 401_2015_1515_MOESM3_ESM.docx]

**Supplementary Table 2.** **Reviewer’s evaluation of presumptive non-CTE tauopathies**

| **Age** | **Sex** | **Head Injury** | **SDx** | **Reviewers Responses** | | | | | | |
| --- | --- | --- | --- | --- | --- | --- | --- | --- | --- | --- |
| 70-75 | M | SMTBI | AD | AD | AD | AD | AD | AD | AD | AD |
| 90-95 | F | SMTBI | AD | AD | AD | AD | AD | AD | AD | AD |
| 66-70 | M | UK | AD | AD | AD | AD | AD | AD | AD | AD |
| 80-85 | F | UK | AD | AD | AD | AD | AD | AD | AD | CTE  AD |
| 90-95 | F | UK | AD | AD | AD | AD | AD | AD | CTE  AD | FTLD-TDP |
| 76-80 | M | UK | CBD | CBD | CBD | CBD | CBD | CBD | CBD | CBD  CTE |
| 56-60 | M | None | CBD | CBD | CBD | CBD | CBD | CBD | PSP | PSP |
| 100-105 | F | UK | PART | PART | PART | PART | PART | PART | AGD | AGD |
| 90-95 | F | UK | PART | PART | PART | PART | PART | PART | PART | AGD |
| 80-85 | F | UK | PSP | PSP | PSP | PSP | AD AGD | AGD | GPDC | ? |
| 76-80 | M | UK | PSP | PSP | PSP | PART | CTE ADC | CTE AD | AD | ? |
| 70-75 | M | UK | AGD | AGD | AGD ADC | CTE AD | PART | PART | FTLD-Pi | PSP |
| 90-95 | F | SMTBI | AGD | AGD | AGD | AD | CTE AD | PSP | GPDC | MSA |
| 76-80 | M | TBI | GPDC | GPDC | GPDC | GDPC | PSP | PSP | PSP | CTE PART |
| 76-80 | F | UK | GPDC | CTE ADC | CTE ADC | CTE AD | CTE AD HS | CTE AD HS | FTLD-Pi | PSP  AD |
| Abbreviations: AD Alzheimer’s disease, ADC Changes of Alzheimer’s disease, AGD Argyrophilic grain disease, CBD Corticobasal degeneration, FTLD-TDP Frontotemporal Lobar Degeneration with TDP-43 proteinopathy, FTLD-Pi Frontotemporal Lobar Degeneration- Pick’s, GPDC Guamanian Parkinson’s Dementia Complex, HS Hippocampal Sclerosis, LBD Lewy body disease, MSA Multiple System Atrophy, PART Primary age-related tauopathy, PSP Progressive supranuclear palsy, SDX Submitted Diagnosis, SMTBI Single mild traumatic brain injury (concussion), SDX Submitted diagnosis, TBI Single Moderate-Severe TBI, ? Uncertain, UK Unknown.  Red text indicates discrepancies with submission diagnosis. | | | | | | | | | | |
